# From Quantity to Reactivity: Advancing Kinetic‐Based Antioxidant Testing Methods for Natural Compounds and Food Applications

**DOI:** 10.1111/1541-4337.70229

**Published:** 2025-07-15

**Authors:** Rajat Suhag, Lucrezia Angeli, Matteo Scampicchio, Giovanna Ferrentino

**Affiliations:** ^1^ Faculty of Agricultural, Environmental and Food Sciences Free University of Bozen‐Bolzano, Piazza Università Bolzano Italy

**Keywords:** antioxidant activity, food preservation, inhibition constant, radical scavenging, real‐time methods

## Abstract

Traditional antioxidant testing methods primarily focus on quantifying antioxidant capacity but fail to capture their reactivity and effectiveness over time. Kinetic‐based methods provide a deeper understanding of antioxidant performance by assessing reaction rates and inhibition mechanisms. This review discusses the advanced kinetic‐based antioxidant testing methods, including the integration of kinetic modeling in traditional assays like 1,1‐diphenyl‐2‐picrylhydrazyl (DPPH) and oxygen radical absorbance capacity, along with inhibited autoxidation methods based on isothermal calorimetry, oxygen uptake, and differential photocalorimetry (DPC). The principles, advantages, and limitations of these methods are discussed, along with their applications. Additionally, challenges related to instrumentation, standardization, and practical implementation are highlighted. Various kinetic‐based antioxidant testing methods have been developed, each with its own set of advantages and limitations. Despite these differences, all these methods share one significant advantage: the ability to provide detailed kinetic information on antioxidant behavior. Continuous monitoring of antioxidant reactivity enables a deeper understanding of how these compounds function in inhibiting oxidation. Furthermore, most of these methods allow testing in real food‐based oxidizable substrates, enhancing their relevance for food applications. Although techniques such as oxygen uptake and DPC may be limited by throughput capacity, methods like isothermal calorimetry and oxidizable substrate monitoring offer high‐throughput capabilities, making them suitable for large‐scale screening. This review presents a range of kinetic‐based antioxidant testing methods that can be chosen and applied according to specific experimental requirements and convenience, providing flexibility to address various food‐related oxidation challenges.

## 1 Introduction

Oxidation is a common challenge for foods rich in fats and oils, including meat, dairy products, vegetable oils, and baked goods. Unsaturated fatty acids are particularly susceptible to oxidative degradation due to their lower stability compared to saturated fatty acids. Heat‐intensive processing methods, such as frying and cooking, accelerate this process, leading to the formation of oxidation products like aldehydes, ketones, peroxides, and epoxides (Garg et al. 2022; Laguerre et al. 2020). These compounds have undesirable characteristics, including unpleasant odors (e.g., the rancid smell of oxidized fats), altered physicochemical properties compared to the original lipids, and, most importantly, toxic effects due to their high electrophilic reactivity (Helberg and Pratt 2021). Beyond sensory deterioration, lipid oxidation can reduce nutritional value by degrading essential fatty acids and fat‐soluble vitamins, generating potentially carcinogenic and pro‐inflammatory compounds that pose health risks. Economically, oxidative spoilage results in significant product losses throughout processing and storage, undermining food safety and profitability. Therefore, preventing or delaying oxidative degradation is crucial not only to maintain quality and extend shelf life but also to ensure consumer safety and minimize economic losses (Shahidi and Zhong 2010; Wang et al. 2023). Antioxidants are commonly added in effective concentrations to inhibit oxidative degradation and extend the shelf life of food products under normal storage conditions. Their role and mechanisms of action have led to extensive research.

**SCHEME 1:**
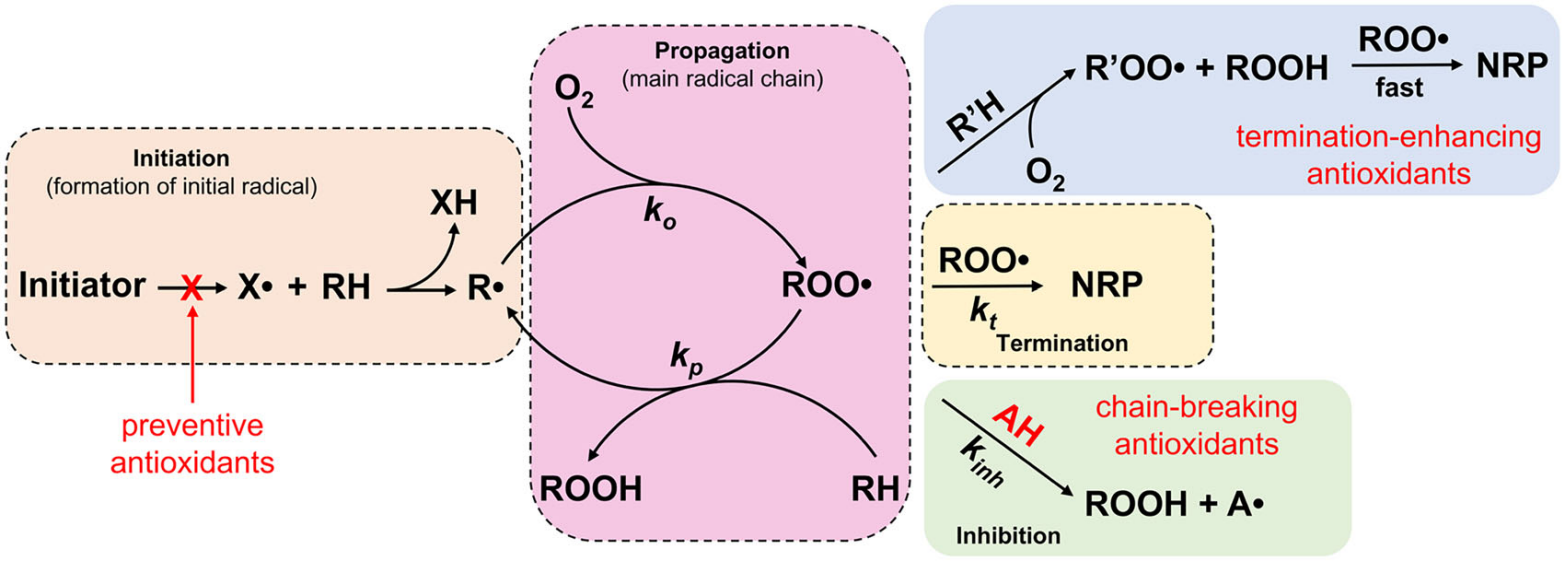
General mechanism of oxidation and interference pathways for preventive, chain‐breaking, and termination‐enhancing antioxidants. RH—oxidizable substrate, R•—alkyl radical, ROO•—peroxyl radical, ROOH—hydroperoxides, AH—antioxidant, NRP—non‐radical products, *k_o_*—rate constant of formation of peroxyl radical is very fast near the diffusion‐controlled limit (≈10^9^ M^−1^ s^−1^), *k_p_*—rate constant of propagation reaction, *k*_inh_—rate constant of inhibition reaction, and *k_t_*—rate constant of termination reaction. *Source*: Adapted from Amorati and Valgimigli (2018) with permission from American Chemical Society.

Antioxidants comprise a diverse group of compounds ranging from small molecules to complex systems that, when added in small quantities (typically less than 1%) relative to an oxidizable substrate, can delay, slow, or prevent its oxidation (Scheme 1) (Burton and Ingold 1986; Halliwell and Gutteridge 2015; Valgimigli and Pratt 2012). Depending on their mechanism of action, antioxidants are categorized into “preventive,” “chain‐breaking” (Ingold 1961; Ingold and Pratt 2014), and “termination‐enhancing” antioxidants, a newer class recently introduced by the group of Valgimigli and Amorati (Amorati et al. 2013; Valgimigli 2023). Preventive antioxidants inhibit the initiation of oxidation reactions by inhibiting the formation of free radicals and blocking the formation of alkyl radicals, with examples including catalase (EC 1.11.1.6) and metal chelators like phytic acid (Ingold and Pratt 2014; Valgimigli and Pratt 2012). Chain‐breaking antioxidants, the largest class of small‐molecule antioxidants (e.g., phenols), are known for interfering with the propagation of oxidation neutralizing alkylperoxyl radicals. Their effectiveness depends entirely on kinetics (*k*_inh_), requiring faster reactions with peroxyl radicals than the propagation rate (i.e., *k*_inh_ >> *k_p_*) (Amorati and Valgimigli 2015; Ingold and Pratt 2014; Valgimigli 2023). Termination‐enhancing antioxidants, like some non‐phenolic terpenes, facilitate the termination of radical reactions. Certain hydrocarbons, aldehydes, and terpenes (e.g., γ‐terpinene, limonene, linalool, and citral) undergo rapid autoxidation when exposed to radicals, promoting chain termination and slowing overall degradation. Although less efficient than phenolic chain‐breakers, these compounds exhibit termination‐enhancing antioxidant behavior (Baschieri et al. 2017; de Sousa et al. 2023; Guo, Pizzol, et al. 2021; Pan et al. 2024).

The ability of antioxidants to prolong the shelf life of food products without compromising their sensory or nutritional qualities has led to the development of thousands of potential compounds over the past century, driving the innovation and adoption of diverse testing methods. Common examples include oxygen radical absorbance capacity (ORAC), cupric‐ion‐reducing antioxidant capacity (CUPRAC), ferric reducing antioxidant power (FRAP), Folin–Ciocalteu's reagent reducing ability, scavenging effects in relation to 1,1‐diphenyl‐2‐picrylhydrazyl (DPPH), and 2,2′‐azino‐bis(3‐ethylbenzothiazoline‐6‐sulphonic acid) (ABTS) (Granato et al. 2018; Gulcin 2020; Munteanu and Apetrei 2021; Yang et al. 2020).

Recent critiques point out significant shortcomings in these assays, as they exclude peroxyl radicals and are carried out in polar organic solvents lacking food‐based oxidizable substrates. These limitations render the resulting numerical values or antioxidant rankings irrelevant in physical or chemical terms, diminishing their applicability to real food systems (Asma et al. 2024; Granato et al. 2018; Laguerre et al. 2007). Additionally, Schaich et al. (2015) emphasized that the initial focus on simply identifying natural extracts with strong antioxidant activity has evolved into a need for a deeper understanding of the mechanisms through which these antioxidants function. They highlighted the importance of prioritizing fundamental chemistry in antioxidant assays over simple or rapid testing methods. This shift is necessary to uncover the radical‐quenching mechanisms of various phenolic compounds, determine rate constants and reaction specificity with different radicals, and identify synergistic or antagonistic interactions among antioxidant classes. Such insights can then be applied to optimize the use of natural antioxidants in both medical therapies and food preservation.

Recently, several kinetic‐based approaches for studying antioxidant reactivity have been developed. Some of these advancements involve modifying existing techniques, such as DPPH (Angeli et al. 2023) and ORAC (Asma et al. 2024), by integrating kinetic models. Others include approaches based on inhibited autoxidation, such as oximetry (Baschieri et al. 2019; Pan et al. 2024), calorimetry (Suhag, Ferrentino, et al. 2024; Suhag, Razem, et al. 2024), and fluorescence‐enabled inhibited autoxidation (Shah et al. 2019; Suhag, Jin, et al. 2024). By incorporating appropriate kinetic analyses, these methods provide valuable real‐time parameters to describe antioxidant activity, such as the induction period length and oxidation rates, both in the presence and absence of antioxidants. A significant advantage of these methods is their ability to utilize real food‐based oxidizable substrates, overcoming the limitations of traditional assays (comparative analysis reported in Table 1). This not only offers deeper insights into antioxidant mechanisms but also facilitates the development of improved and more effective preservative strategies. The aim of this review is to discuss the recently developed kinetic‐based antioxidant testing methods without focusing on conventional antioxidant assays because several studies have discussed their advantages and limitations (Amorati and Valgimigli 2015, 2018; Bibi Sadeer et al. 2020; Granato et al. 2018; Gulcin 2020; Lang et al. 2024; Munteanu and Apetrei 2021; Schaich et al. 2015).

**TABLE 1:** Comparison of traditional and kinetic‐based antioxidant assays.

Traditional antioxidant assays	Kinetic‐based methods
Single‐point analysis	Real‐time analysis providing a continuous stream of data
Carried out in organic solvents	Carried out in real food‐based matrices (e.g., oxidizable substrate monitoring, isothermal calorimetry, oxygen uptake method, and differential photocalorimetry)
Performed in the presence of organic solvents, which may impact the results	Carried out in solvent‐free environment
Use of artificial radicals (e.g., DPPH) that are not found in food products	Involve peroxyl radicals
No clear differentiation between slow‐ and fast‐reacting antioxidants	Differentiate antioxidants based on reactivity

Abbreviation: DPPH, 1,1‐diphenyl‐2‐picrylhydrazyl.

In detail, the review will discuss the mechanism, applications, advantages, and challenges associated with the kinetic‐based methods, providing insights on the study of the antioxidant reactivity and guiding researchers in exploring these advanced approaches. The development of kinetic‐based methods represents a significant step toward transitioning from conventional assays to real‐time kinetic analysis performed on real food matrices. These advancements hold great promise for improving food quality, stability, and shelf life while advancing the field of food preservation.

## 2 Importance of Kinetic‐Based Approaches

Although antioxidants are considered bioactive compounds, their activity differs significantly from that of drugs. Most drugs, whether natural or synthetic, exert their effects through noncovalent binding to specific cellular receptors or enzymes, which alters cellular functions. Their effectiveness is primarily determined by the binding extent, and chemically, this is quantified through an equilibrium constant (Amorati and Valgimigli 2018). In contrast, assessing antioxidant activity involves measuring the rate at which antioxidants react with radicals, such as peroxyl radicals, or how they influence the autoxidation rate of the substrate they protect (Grebowski et al. 2020; Guo, Pizzol, et al. 2021; Helberg and Pratt 2021). For example, a radical‐trapping antioxidant, regardless of its effectiveness, will react completely with peroxyl radicals due to the exothermic nature of the reaction (Amorati and Valgimigli 2018; Hanthorn et al. 2012). The key difference between a modest and an excellent antioxidant lies in the speed of this reaction. Radical‐trapping antioxidants can protect substrates from peroxyl radicals only if their reaction rate is faster than that of the substrate itself (Haidasz et al. 2016; Niki 2010). Therefore, the most critical parameters for quantifying antioxidant activity are the rate constants for radical trapping and the reaction stoichiometry. Stoichiometry defines the quantitative capacity of an antioxidant to neutralize radicals, representing the number of radicals scavenged per molecule. For example, in DPPH kinetics assays, both ascorbic acid and syringic acid exhibit a stoichiometry of 2, indicating that one antioxidant molecule can scavenge two radicals. In contrast, the rate constant reflects the reaction speed, describing how rapidly the antioxidant quenches radicals. Ascorbic acid, with a rate constant, *k* ∼ 21,100 M^−1^ s^−1^, reacts significantly faster than syringic acid (*k* ∼ 400 M^−1^ s^−1^), despite their comparable stoichiometries (Angeli et al. 2023). Although stoichiometry determines the total antioxidant capacity, the rate constant governs kinetic efficiency, a critical factor for protecting fast‐oxidizing substrates, such as unsaturated fatty acids, where rapid radical quenching is essential. It is important to treat these two parameters separately, as combining them can hinder a meaningful comparison of different antioxidants (Amorati and Valgimigli 2015; Y. Guo, Baschieri, et al. 2021; Helberg and Pratt 2021; Niki 2010; Shah et al. 2019).

Apart from the limitations of antioxidant assays discussed earlier, the way in which their data are handled and reported significantly impacts their findings, drawing attention to the role of kinetic analysis. In the following paragraphs, some case studies are reported. Establishing a quantitative framework is the key to improving the antioxidant evaluations. Comparisons between different antioxidants should rely on well‐defined and measurable factors, with kinetic data playing an essential role (Amorati and Valgimigli 2018; Pinchuk et al. 2012).

### 2.1 Case of Oxidation Methods

**FIGURE 1:**
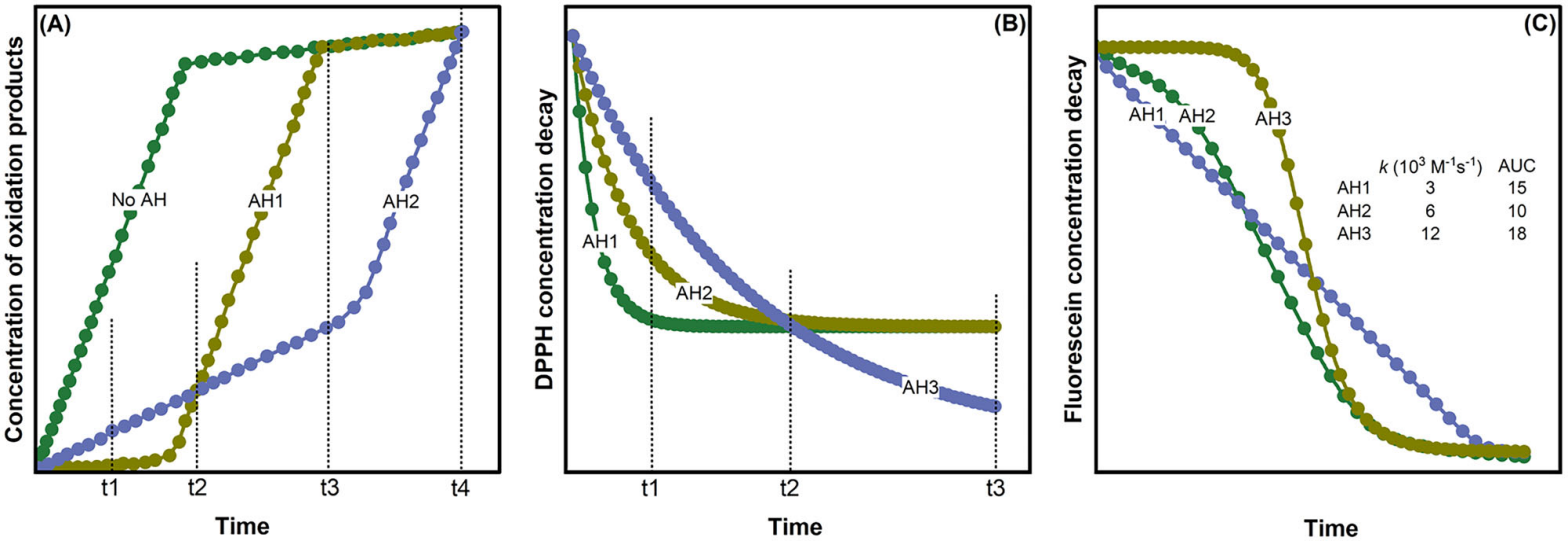
Schematic representation of (A) accumulation of oxidation products during autoxidation of a substrate without any added antioxidants (No AH) and with different antioxidants (AH_1_ and AH_2_); (B) decrease in the DPPH concentration due to scavenging by different antioxidants (AH_1_, AH_2_, and AH_3_); and (C) decay of fluorescein fluorescence in the presence of different antioxidants (AH_1_, AH_2_, and AH_3_) during ORAC assay, where *t*_1_, *t*_2_, *t*_3_, and *t*_4_ represent random time intervals in the assays; *k*—rate constant; and AUC—area under the curve values for different antioxidants in the ORAC assay. DPPH, 1,1‐diphenyl‐2‐picrylhydrazyl.

Often, antioxidants tested in equal concentrations under identical conditions are compared on the basis of oxidation product levels at a specific time point. However, this can be misleading, as the concentration of oxidation products depends on the assay's duration. Figure 1A shows the concentration of the oxidation products as a function of the time. At an early time point (*t*_1_), the concentration of oxidized products is lower in the presence of the antioxidant AH_1_ compared to the antioxidant AH_2_. This indicates that AH_1_ provides more effective protection against peroxidation. However, at time *t*_2_, both antioxidants exhibit a similar concentration of oxidized products, indicating no difference in antioxidant effectiveness. At a later stage, time *t*_3_, the reverse is shown with AH_2_ demonstrating better effectiveness than AH_1_. Finally, once both antioxidants are fully consumed, peroxidation accelerates. The concentration of oxidation products is similar if analyzed at time *t*_4_, regardless of whether the antioxidant is present or not. A measurement performed at this point may falsely imply that neither AH_1_ nor AH_2_ possesses antioxidant properties (Pinchuk et al. 2012).

### 2.2 Case of DPPH Assay

A similar issue arises with the commonly used DPPH assay for testing antioxidant activity. The assay is based on the hydrogen donation from antioxidants leading to the transformation from DPPH• to non‐radical DPPH‐H. DPPH• gives a strong absorption band around 517 nm depending on the solvents. As the non‐radical DPPH‐H is formed, the absorbance decreases, causing the color to turn from purple to yellow. Figure 1B shows a simulated time course of the reaction of three antioxidants with different reactivity toward the DPPH• (*k*_DPPH_) and stoichiometry factor (*n*). It can be observed that the reactivity of the antioxidants with DPPH• is in the order AH_1_ > AH_2_ > AH_3_. Generally, the results of the DPPH assay are expressed as EC_50_ or % DPPH loss after a fixed time (Schaich et al. 2015). Considering this single‐point measurement approach, if the % DPPH loss is measured at time *t*_1_ (in Figure 1B), the order is AH_1_ > AH_2_ > AH_3_, whereas when measured at time *t*_2_, all three antioxidants report comparable activity. If measured at time *t*_3_, the order completely changes to AH_1_ ≈ AH_2_ > AH_3_.

The above‐discussed examples highlight the variability in findings when measurements of the antioxidant activity are performed using a single‐point approach. To overcome such variations, it would be valuable to perform full kinetic analysis, providing information on the rate kinetics of the reactivity of the antioxidants with the radicals. This proves once more the clear need for real‐time, kinetic‐based methods to assess antioxidant activity and lipid oxidation.

### 2.3 Case of ORAC Assay

Measurements of the “area under the curve” (AUC), as proposed for evaluating antioxidative effects in several assays (Re et al. 1999; Sadowska‐Bartosz and Bartosz 2022), offer a partial solution to the limitations of single‐point measurements by relying on continuous recording of data. The AUC approach is commonly employed in the ORAC assay, which uses fluorescein (FH) as a fluorescent probe that loses its fluorescence upon reacting with radicals (Asma et al. 2024). AUC integrates the induction time with the total quantity of free radicals reacting with antioxidants (stoichiometry factor, *n*). However, it has several notable limitations (Carvalho et al. 2023). This approach primarily focuses on the total antioxidant quantity, giving less consideration to their reactivity toward radicals, thus failing to capture the kinetics of these interactions (Valgimigli and Pratt 2012). For instance, Figure 1C depicts a simulated ORAC curve over time for three different antioxidants with varying reactivity (AH_3_ > AH_2_ > AH_1_). On the basis of the AUC, AH_3_ appears slightly more effective than AH_1_, but the kinetic analysis reveals that it is four times more effective. Although AUC values suggest AH_3_ is superior to AH_1_, a kinetic analysis allows to distinguish the stoichiometry (induction time) from the rate constant of radical trapping. By combining these two parameters, the AUC method assigns more weight to stoichiometry than reactivity, leading to misleading conclusions about the antioxidant effectiveness (Amorati and Valgimigli 2015).

### 2.4 Limitations of Traditional Methods in Practical Applications

Beyond experimental and data interpretation limitations, a critical consideration related to traditional methods is whether the results obtained by these antioxidant assays translate to real‐world applications. For example, Mohammadi et al. (2024) demonstrated that the antioxidant potential of fruit‐derived phenolic compounds (e.g., catechin, hyperoside, and phlorizin), as determined by traditional assays, poorly correlated with their ability to protect human plasma from oxidative damage. Similarly, Pasqualetti et al. (2021) found no significant correlation between the antioxidant capacity of grapeseed, rosemary leaf, and pomegranate mesocarp extracts and their efficacy in shielding red blood cell (RBC) membranes from UV‐induced oxidative stress. Moreover, Souza et al. (2008) further reported that the phenolic content, ORAC values, and total radical‐trapping antioxidant potential of polyphenol‐rich Amazonian plant extracts did not align with their erythrocyte‐protective effects. Other studies similarly report that traditional antioxidant assays do not always predict real‐world efficacy (Aouachria et al. 2017; Sun et al. 2017).

However, conflicting evidence exists. Some studies show correlations between traditional assay outcomes and biological activity (Corrigan et al. 2023; Dong et al. 2022; Y. Zhang, Li, et al. 2023). This variability underscores the complexity of linking *in* *vitro* antioxidant measurements to antioxidative protection in biological or food systems. Evidently, antioxidant activity involves intricate interactions between intrinsic factors (e.g., food matrix composition) and extrinsic conditions (e.g., bioavailability and metabolic pathways), which cannot be fully captured by simplified chemical reactions used in traditional assays (Granato 2023). Despite these limitations, dismissing traditional assays would be impractical. These tools remain vital as cost‐effective, high‐throughput strategies for identifying potential antioxidant sources, although there are ongoing debates about their biological relevance (Alves et al. 2017; Granato et al. 2018).

## 3 Kinetic‐Based Antioxidant Testing Methods

### 3.1 Spectrophotometric Assays

Despite the limitations of commonly used spectrophotometric assays, such as DPPH, ABTS, and ORAC, they remain invaluable due to their low cost, high throughput, ease of use, and minimal need for sensitive equipment. These assays are widely applied to assess the antioxidant capacity in both isolated compounds and mixed extracts from complex food matrices (Bibi Sadeer et al. 2020; Granato et al. 2018). Moreover, they serve as effective screening methods, providing initial insights into the antioxidant capacity (Schaich et al. 2015) and helping identify potential dietary antioxidant sources (Alves et al. 2017). To enhance the utility of these assays and provide information on the antioxidants reactivity rather than just their capacity, some modifications incorporating kinetic analysis have been introduced. Additionally, innovative approaches have been developed to evaluate the reactivity of antioxidants in food‐based oxidizable substrates using microplate readers. The following sections will discuss in detail these advancements, which are also summarized in Table 2.

**TABLE 2:** Application of different kinetic‐based methods to determine the reactivity of antioxidants.

Method	Application	Findings	Reference
DPPH stopped‐flow kinetic	Standard antioxidants	Ascorbic acid: *k*_1_ = 21,100 ± 570 M^−1^ s^−1^ Rutin: *k*_1_ = 3070 ± 300 M^−1^ s^−1^; *k*_2_ = 60.0 ± 4.3 M^−1^ s^−1^ Ellagic acid: *k*_1_ = 2900 ± 160 M^−1^ s^−1^; *k*_2_ = 18.7 ± 1.4 M^−1^ s^−1^ Quercetin: *k*_1_ = 2380 ± 81 M^−1^ s^−1^; *k*_2_ = 52.5 ± 1.3 M^−1^ s^−1^ Tannic acid: *k*_1_ = 1730 ± 64 M^−1^ s^−1^; *k*_2_ = 21.8 ± 1.8 M^−1^ s^−1^ Epicatechin: *k*_1_ = 778 ± 43 M^−1^ s^−1^; *k*_2_ = 30.3 ± 1.8 M^−1^ s^−1^ Trolox: *k*_1_ = 657 ± 47 M^−1^ s^−1^ α‐tocopherol: *k*_1_ = 601 ± 11 M^−1^ s^−1^ Syringic acid: *k*_1_ = 401 ± 24 M^−1^ s^−1^; *k*_2_ = 14.9 ± 1.3 M^−1^ s^−1^ Catechin: *k*_1_ = 252 ± 5 M^−1^ s^−1^; *k*_2_ = 25.8 ± 1.1 M^−1^ s^−1^ Protocatechuic acid: *k*_1_ = 172 ± 13 M^−1^ s^−1^ Phloretin: *k*_1_ = 45.1 ± 2.6 M^−1^ s^−1^	Angeli et al. (2023)
	Fruit extracts	Strawberry: *k*_1_ = 4880 ± 100 M^−1^ s^−1^; *k*_2_ = 17.1 ± 0.21 M^−1^ s^−1^ Kiwi: *k*_1_ = 3550 ± 80 M^−1^ s^−1^ Grapefruit: *k*_1_ = 2260 ± 59 M^−1^ s^−1^ Peach: *k*_1_ = 694 ± 6.3 M^−1^ s^−1^ Apricot: *k*_1_ = 297 ± 19 M^−1^ s^−1^ Apple: *k*_1_ = 161 ± 16 M^−1^ s^−1^ Red plum: *k*_1_ = 134 ± 5.1 M^−1^ s^−1^	
	Apple varieties	Majda: *k*_1_ = 5540 ± 390 M^−1^ s^−1^; *k*_2_ = 150 ± 5 M^−1^ s^−1^ Golden delicious: *k*_1_ = 231 ± 9 M^−1^ s^−1^; *k*_2_ = 54.7 ± 2 M^−1^ s^−1^ R201: *k*_1_ = 215 ± 9 M^−1^ s^−1^; *k*_2_ = 51.3 ± 2 M^−1^ s^−1^	Angeli et al. (2024)
	Maillard reaction products	Maillard reaction products: *k*_1_ = 6000 ± 300 M^−1^ s^−1^ Ascorbic acid: *k*_1_ = 14,450 ± 750 M^−1^ s^−1^ Trolox: *k*_1_ = 1770 ± 90 M^−1^ s^−1^	Bolchini et al. (2025)
ORAC kinetics	Standard antioxidants	Trolox: *k*_5_ = 400 × 10^3^ M^−1^ s^−1^ Caffeic acid: *k*_5_ = 53 ± 5 × 10^3^ M^−1 ^s^−1;^ *k*_6_ = 2.8 ± 0.4 M^−1^ s^−1^; AUC = 3.9 ± 0.1; *n* = 4.3 ± 0.6 Sinapic acid: *k*_5_ = 60 ± 2 × 10^3^ M^−1^ s^−1;^ *k*_6_ = 6.9 ± 0.2 M^−1^ s^−1^; AUC = 2.0 ± 0.1; *n* = 1.0 ± 0.2 Ferulic acid: *k*_5_ = 30 ± 2 × 10^3^ M^−1^ s^−1^; *k*_6_ = 2.4 ± 0.3 M^−1^ s^−1^; AUC = 3.3 ± 0.2; *n* = 1.5 ± 0.3 *p*‐coumaric acid: *k*_5_ = 20 ± 1 × 10^3^ M^−1^ s^−1^; *k*_6_ = 1.5 ± 0.2 M^−1^ s^−1^; AUC = 4.0 ± 0.4; *n* = 2.0 ± 0.2 Chlorogenic acid: *k*_5_ = 61 ± 4 × 10^3^ M^−1^ s^−1^; *k*_6_ = 3.0 ± 0.3 M^−1^ s^−1^; AUC = 3.8 ± 0.1; *n* = 4.0 ± 0.5	Asma et al. (2024)
	Fruit juice extracts	Order of *k*_5_: lemon ∼ mango > orange ∼ grape ∼ pomegranate > pineapple > apple	
Oxidizable substrate monitoring	Synergistic interactions with PMC (3 µM)	PMC + γ‐terpinene (6.3 mM): *k*_inh_ = 1.2 ± 0.3 M^−1 ^s^−1^, *n* = 2.3 ± 0.1 PMC + γ‐terpinene (15.4 mM): *k*_inh_ = 1.3 ± 0.1 M^−1^ s^−1^, *n* = 2.7 ± 0.1 PMC + γ‐terpinene (29.4 mM): *k*_inh_ = 1.2 ± 0.2 M^−1^ s^−1^, *n* = 3.3 ± 0.3 PMC + quercetin (3 µM): *k*_inh_ = 1.1 ± 0.3 M^−1^ s^−1^, *n* = 2.4 ± 0.1 PMC + quercetin (3 µM): *k*_inh_ = 1.2 ± 0.2 M^−1^ s^−1^, *n* = 2.7 ± 0.2 PMC + quercetin (3 µM): *k*_inh_ = 1.2 ± 0.1 M^−1^ s^−1^, *n* = 3.0 ± 0.2 PMC + caffeic acid (10 µM): *k*_inh_ = 1.3 ± 0.3 M^−1^ s^−1^, *n* = 2.3 ± 0.2 PMC + caffeic acid (20 µM): *k*_inh_ = 1.2 ± 0.2 M^−1^ s^−1^, *n* = 2.6 ± 0.1 PMC + caffeic acid (30 µM): *k*_inh_ = 1.1 ± 0.3 M^−1^ s^−1^, *n* = 2.8 ± 0.2	Suhag, Jin, et al. (2024)
Oxygen uptake method	Essential oils	*Thymus vulgaris*: *k*_inh_ = 1.5 ± 0.3 × 10^4^ M^−1^ s^−1^ *Origanum vulgare*: *k*_inh_ = 1.3 ± 0.1 × 10^4^ M^−1^ s^−1^ *Satureja hortensis*: *k*_inh_ = 1.1 ± 0.3 × 10^4^ M^−1^ s^−1^ *Eugenia caryophyllus*: *k*_inh_ = 5.0 ± 0.1 × 10^3^ M^−1^ s^−1^ *Cinnamomum zeylanicum*: *k*_inh_ = 4.9 ± 0.3 × 10^3^ M^−1^ s^−1^	Guo, Pizzol, et al. (2021)
		*Juniperus oxycedrus*: *k*_inh_ = 1.2 ± 0.2 × 10^4^ M^−1^ s^−1^ *Syzygium aromaticum: k*_inh_ = 7.0 ± 0.5 × 10^3^ M^−1^ s^−1^ *T. vulgaris*: *k*_inh_ = 1.5 ± 0.3 × 10^4^ M^−1^ s^−1^ *Thymbra capitata*: *k*_inh_ = 1.5 ± 0.2 × 10^4^ M^−1^ s^−1^ *Betula alba*: *k*_inh_ = 1.0 ± 0.1 × 10^4^ M^−1^ s^−1^ *Pimenta racemosa*: *k*_inh_ = 8.9 ± 0.5 × 10^3^ M^−1^ s^−1^ *Satureja montana*: *k*_inh_ = 1.4 ± 0.2 × 10^4^ M^−1^ s^−1^	Pan et al. (2024)
	Natural antioxidants	In chlorobenzene: Magnolol: *k*_inh_ = 6.1 ± 0.5 × 10^4^ M^−1^ s^−1^, *n* = 2.0 ± 0.4 Honokiol: *k*_inh_ = 3.8 ± 0.4 × 10^4^ M^−1^ s^−1^, *n* = 2.2 ± 0.1 In acetonitrile: Magnolol: *k*_inh_ = 6.0 ± 0.7 × 10^3^ M^−1^ s^−1^, *n* = 2.1 ± 0.2 Honokiol: *k*_inh_ = 9.5 ± 1.5 × 10^3^ M^−1^ s^−1^, *n* = 3.7 ± 0.3	Amorati et al. (2015)
	Acylated anthocyanins from sprouts of *Raphanus sativus* cv. Sango	*k*_inh_ = 3.8 ± 0.7 × 10^4^ M^−1^ s^−1^	Matera et al. (2015)
Isothermal calorimetry	Mayonnaise	Apple seed oil mayonnaise: *k*_inh_ = 3.9 ± 0.3 × 10^3^ M^−1^ s^−1^ Sunflower oil mayonnaise: *k*_inh_ = 5.7 ± 0.2 × 10^3^ M^−1^ s^−1^ Corn oil mayonnaise: *k*_inh_ = 0.8 ± 0.1 × 10^3^ M^−1^ s^−1^ Grapeseed oil mayonnaise: *k*_inh_ = 2.0 ± 0.1 × 10^3^ M^−1^ s^−1^ Extra virgin olive oil mayonnaise: *k*_inh_ = 0.4 ± 0.1 × 10^3^ M^−1^ s^−1^	Suhag, Ferrentino, et al. (2024)
Differential photocalorimetry	Vegetable seed oils	Ferulic acid: *k*_inh_ = 7.2 × 10^4^ M^−1^ s^−1^ Oxidative stability of oils: Rice bran > corn > soybean > sunflower oil	Suhag, Razem, et al. (2024)

Abbreviations: AUC, area under the curve; DPPH, 1,1‐diphenyl‐2‐picrylhydrazyl; PMC, 2,2,5,7,8‐pentamethyl‐6‐chromanol; ORAC, oxygen radical absorbance capacity.

#### 3.1.1 Stopped‐Flow DPPH Kinetic Method

**FIGURE 2:**
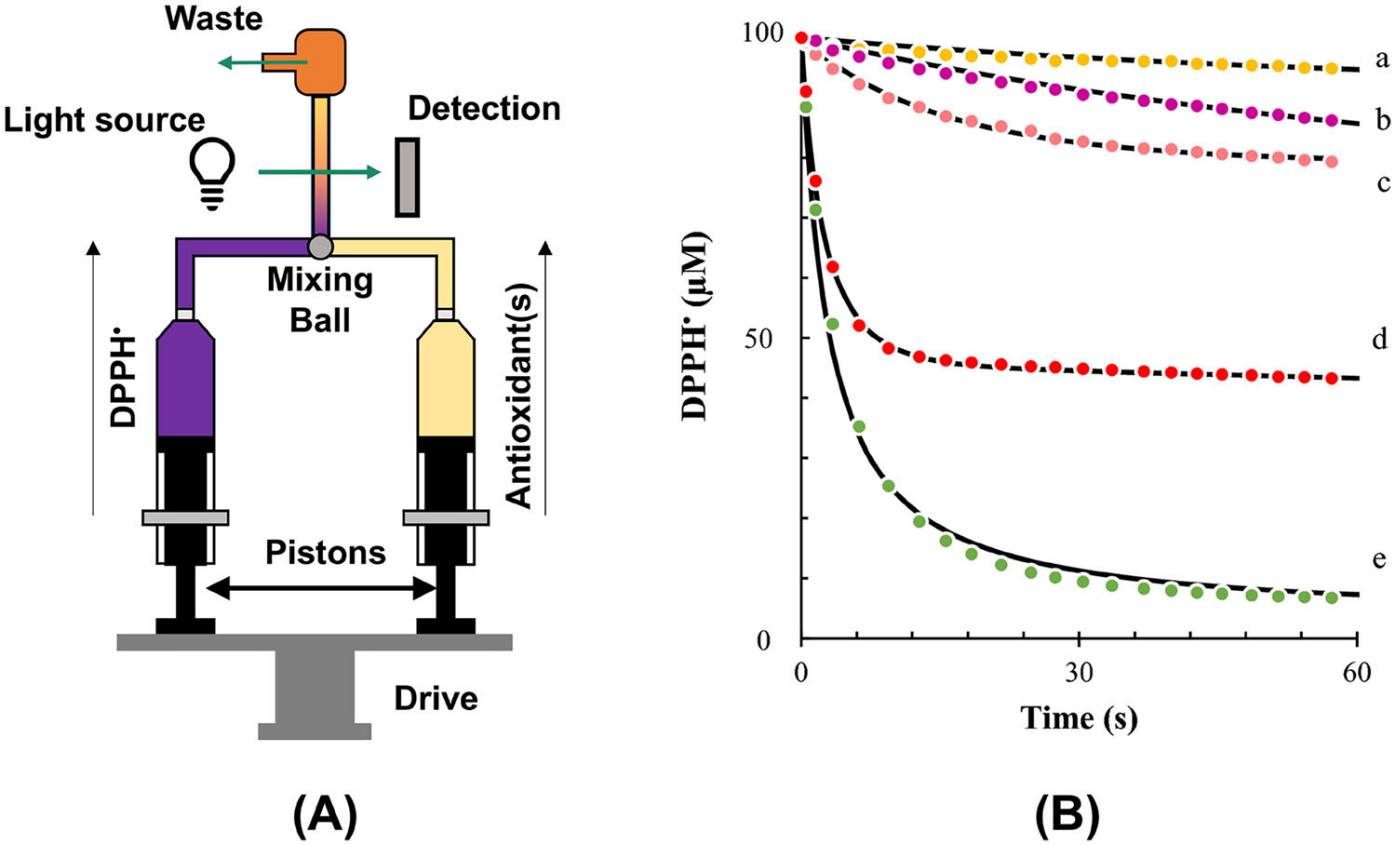
(A) Schematic representation of a stopped‐flow system consisting of two syringes with pistons that push the two reagents (DPPH• and antioxidant solution) into a mixing chamber and then into the quartz flow cell, driven simultaneously by the same driving motor, and (B) kinetic curves and fittings of the reaction between 100 µM of DPPH• and (a) apple, (b) red plum, (c) peach, (d) strawberry, and (e) kiwi extracts, standardized to 30 µM gallic acid equivalent (GAE). DPPH, 1,1‐diphenyl‐2‐picrylhydrazyl. *Source*: Reprinted from Angeli et al. (2023) under a Creative Commons Attribution 4.0 International License.

The stopped‐flow method, known for its ability to capture rapid reactions, becomes even more effective when coupled with a rapid‐scanning UV/Vis spectrophotometer for analyzing reaction mechanisms (Xie and Schaich 2014; K. Zhang et al. 2000). Figure 2A illustrates a schematic representation of a stopped‐flow system. This setup employs a pneumatic drive to quickly mix two solutions contained in separate syringes—one holding the DPPH solution and the other the antioxidant solution. The experiment begins by simultaneously activating the pistons of both syringes, forcing the solutions to mix in the mixing chamber. The mixed solutions are then transferred to the cuvette cell with a maximum delay of 6 ms. The resulting absorbance of the reaction mixture is recorded every 18 ms at a wavelength of 515 nm using the UV/Vis spectrophotometer (Angeli et al. 2023).

The stopped‐flow DPPH kinetic method enables not only the determination of total antioxidant capacity but also the stoichiometry and rate constants of the reactions involved. Because the reactivity depends on the type of solvent used and the initial concentrations of both the radical and the antioxidant mixture, careful standardization of the method is required (Foti et al. 2004; Xie and Schaich 2014). Angeli et al. (2023) investigated the electron transfer reactions using methanol as a solvent under conditions with an excess of DPPH• to determine the stoichiometry. Under these conditions, antioxidants react with DPPH• as described in Equation (1). Specifically, DPPH• reacts with antioxidant (AH) to form the reduced form of DPPH• (DPPH‐H) and the oxidized form of the antioxidants (A•). The oxidized antioxidant (A•) subsequently participates in a secondary reaction, referred to as the side reaction (Equation 2), with the remaining DPPH•, producing unknown products. This secondary reaction explains why the reaction does not reach a plateau. These products have been characterized for some antioxidant molecules using LC–MS (Angeli et al. 2021):

(1) $$\mathrm{DPPH}•+\mathrm{AH}\overset{k_{1}}{\to }\mathrm{DPPH}-H+A\;$$

(2) $$A•+\;\mathrm{DPPH}•\overset{k_{2}}{\to }\mathrm{products}$$

Figure 2B illustrates that the DPPH• concentration decreases rapidly within the first few seconds, followed by a slower decline over several minutes due to the side reaction (Angeli et al. 2021).

The kinetic data can be simulated and fitted using Copasi software (Hoops et al. 2006). The DPPH• consumption simulations are generated by solving a system of differential equations based on the law of mass action applied to Equations (1) and (2). The optimal kinetic parameters (*k*_1_ and *k*_2_) and reaction stoichiometry (*n*) are determined by iteratively minimizing the sum of squared errors between the experimental and simulated data. Specifically, the stoichiometric factor is calculated as the ratio of the optimal concentration determined by Copasi to the known concentration added to the reaction mixture (Angeli et al. 2023).

##### 3.1.1.1 Applications of Stopped‐Flow DPPH Kinetic Method

###### 3.1.1.1.1 Standard Antioxidants

The stopped‐flow method has been effectively used to determine the reactivity of standard antioxidants as well as various food samples. Among the standard antioxidants tested, ascorbic acid exhibited the highest reactivity (*k*_1_ = 21,100 M^−1^ s^−1^, 10 µM) with no contribution from side reactions (*k*_2_). Other antioxidants demonstrated significantly lower rate constants, from 10 to 500 times lower than that of ascorbic acid (Table 2). Similar to ascorbic acid, compounds such as Trolox, α‐tocopherol, protocatechuic acid, and phloretin showed no side reaction. In contrast, rutin, ellagic acid, quercetin, tannic acid, epicatechin, catechin, and syringic acid exhibited side reactions (Angeli et al. 2023). These side reactions are attributed to interactions between antioxidant radicals and DPPH• radicals, forming new stable, non‐radical complexes that slow down DPPH• consumption (Angeli et al. 2021; Foti and Daquino 2006).

###### 3.1.1.1.2 Fruit Juices and Apple Extracts

The application of the stopped‐flow method to fruit extracts provided insights into the reactivity of antioxidants in complex mixtures. Among the tested extracts, strawberry exhibited the highest reactivity (*k*_1_ = 4880 M^−1^s^−1^), followed by kiwi > grapefruit > peach > apricot > apple > red plum (Figure 2B). Of these, only strawberry showed a side reaction (*k*_2_ = 17.1 M^−1^s^−1^) (Angeli et al. 2023). Additionally, the stopped‐flow approach offered valuable information about the reactivity of antioxidants in different apple varieties. The anti‐browning variety “Majda” exhibited a *k*_1_ value 24 times higher than those of “Golden Delicious” and “R201” varieties. Higher *k*_1_ value for the “Majda” apple variety was linked to the presence of very reactive antioxidants, mainly ascorbic acid, rather than a high amount of phenolic compounds. Notably, although the classical DPPH• assay failed to detect significant differences among these apple varieties, the kinetic‐based stopped‐flow method enabled better discrimination of antioxidants based on their reactivity (Angeli et al. 2024).

###### 3.1.1.1.3 Maillard Reaction Products

Bolchini et al. (2025) employed stopped‐flow DPPH kinetics to assess the reactivity of antioxidants in Maillard reaction products within a glucose–glycine model system. The reactivity constant of the Maillard reaction products was approximately half compared to the one of ascorbic acid but nearly three times higher than that of Trolox, indicating significant reactivity. However, in terms of antioxidant capacity (i.e., stoichiometry), the Maillard reaction products exhibited a stoichiometric value (*n*) of approximately 1.8–2.0, similar to those of ascorbic acid and Trolox.

##### 3.1.1.2 Advantages and Limitations of Stopped‐Flow DPPH Kinetics

The kinetic‐based stopped‐flow DPPH method offers a significant advantage in capturing rapid reactions, often completed within seconds, making it particularly effective for studying fast‐reacting antioxidants. By incorporating kinetic data modeling, the method enables precise determination of absolute rate constants while distinguishing between primary and side reactions, thus providing deeper insights into reaction dynamics. It is especially valuable for analyzing complex mixtures, such as food extracts, by delivering detailed kinetic parameters that conventional endpoint assays cannot achieve. The real‐time approach enhances the understanding of antioxidant mechanisms, including synergistic or antagonistic interactions in multi‐component systems, making it an essential tool for optimizing antioxidant applications in food preservation.

However, certain considerations are necessary when applying the stopped‐flow method to study antioxidant reactivity. The choice of the reaction solvent significantly affects the reaction pathway and the rate of DPPH• scavenging (Goujot et al. 2019; Litwinienko and Ingold 2007; Shojaee et al. 2022). For instance, gallic acid and methyl gallate exhibited different reactivity depending on the nature of the solvent used during the reaction, with gallic acid having better reactivity than methyl gallate in alcoholic solvents (e.g., ethanol and methanol) and in acetonitrile. Conversely, methyl gallate performed better in polar aprotic solvents (e.g., acetone, ethyl acetate, and tetrahydrofuran). The highest reactivity values for both antioxidants were observed in ethanol and methanol (Shojaee et al. 2022). In hydrogen bond–donating solvents, the reaction mechanism shifts from the slower H‐atom transfer (HAT) pathway to the faster single‐electron transfer pathway due to partial ionization of phenols (Foti et al. 2004; Jodko‐Piórecka et al. 2017; Nenadis and Tsimidou 2017). Methanol and other polar organic solvents are commonly used in DPPH assays due to the radicals high solubility in these media. However, adaptations to aqueous systems, though challenging, are feasible and have been explored (Vinci et al. 2022). Such adaptations often face issues like reduced DPPH• solubility and altered radical stability. Introducing water into the reaction medium disrupts intermolecular interactions (e.g., hydrogen bonding) and promotes HAT, accelerating the reaction for compounds capable of donating hydrogen atoms. In contrast, electron transfer mechanisms are pH‐dependent: Reaction rates increase under alkaline conditions due to greater ionization, whereas HAT remains largely unaffected by pH. To identify the predominant reaction pathway of a test compound, researchers can compare its reactivity with DPPH• in two systems: (1) pure methanol and (2) a 50% methanol–water mixture, where the aqueous phase is buffered across a pH gradient, acidic to alkaline (Karagecili et al. 2023; Schaich et al. 2015).

Furthermore, to ensure reproducibility in the DPPH• method, it is crucial to report the antioxidant concentration, as the rate constant is influenced by reactant concentration (Campos et al. 2012; Foti et al. 2004). For higher antioxidant concentrations, the rate constants are lower (Angeli et al. 2021, 2023) because the formation of phenolic anions—the reactive species—is inhibited, thereby slowing down the overall electron transfer process (Foti 2015). Additionally, kinetic modeling must account for the side reaction. Neglecting side reactions may lead to an underestimation of the rate constants and incorrect estimation of stoichiometry factors, resulting in misleading conclusions (Angeli et al. 2021, 2023; Goujot et al. 2019; Mishra et al. 2012).

#### 3.1.2 ORAC Kinetics

The ORAC assay is a widely used method for assessing the antioxidant capacity of food and biological samples. It employs fluorescein (FH) as a fluorescent probe, which loses fluorescence upon reacting with oxygen radicals produced by the free radical initiator AAPH (2,2′‐azobis(2‐methylpropionamidine) dihydrochloride) (Apak 2019; Cao et al. 1993; Schaich et al. 2015). Although the relevance of ORAC values as indicators of antioxidant capacity remains a topic of discussion (Andrewes 2022; Brainina et al. 2019; Granato et al. 2018), the assay continues to be appreciated for its utility in comparing the radical‐scavenging potential of antioxidants (Apak et al. 2016b; Asma et al. 2024; Carvalho et al. 2023).

**FIGURE 3:**
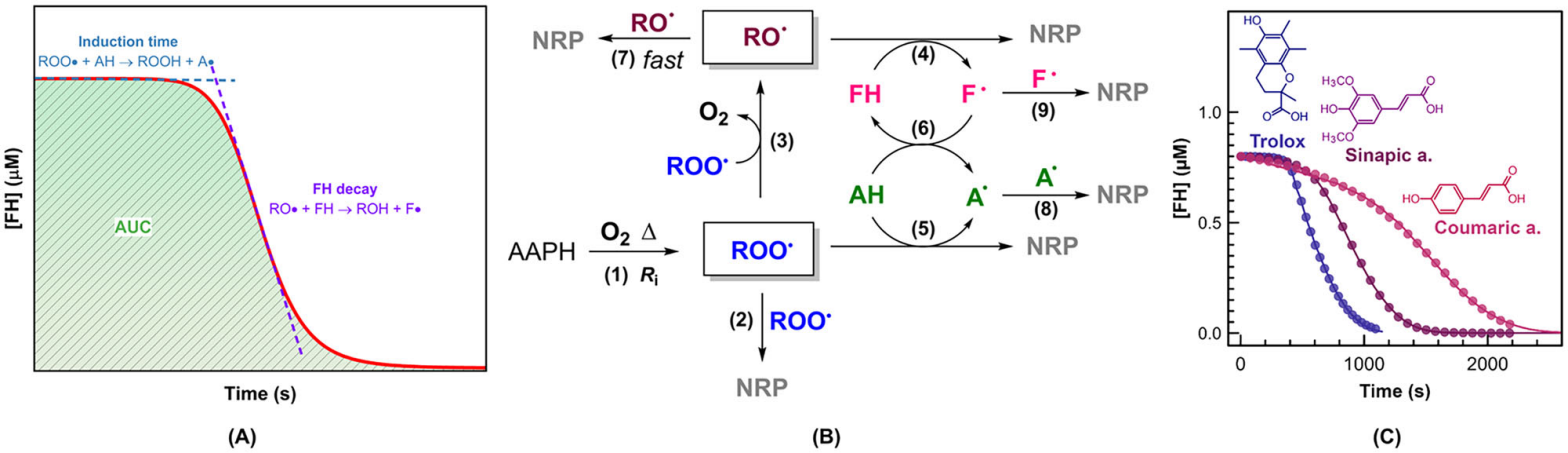
(A) Schematic representation of fluorescein (FH) consumption versus time in ORAC assay. Induction time corresponds to the intersection of the two tangents, (B) scheme of the reaction mechanism describing the ORAC assay, and (C) fluorescein (0.8 µM) bleaching curves in the presence of Trolox, sinapic acid, and *p*‐coumaric acid, each 5 µM, and AAPH (100 mM) at pH 7.0 and 37°C. AAPH is the free radical initiator 2,2′‐azobis(2‐methylpropionamidine) dihydrochloride; RO•—alkoxyl radical; ROO•—peroxyl radical; AH—antioxidant; FH—fluorescein; A• and F•—oxidized forms of antioxidant and fluorescein, respectively, and NRP—non‐radical product. *Source*: (B) and (C) reprinted from Asma et al. (2024) under a Creative Commons Attribution 4.0 International License.

As noted earlier (Section 2), the primary approaches for determining ORAC values and assessing the radical‐scavenging activity of antioxidants include the calculation of the net AUC (Ou et al. 2013) and the induction times (Carvalho et al. 2023; Güçlü et al. 2014). Recent efforts have been made to incorporate kinetic and mechanistic features into ORAC assay to increase its utility and significance. Carvalho et al. (2023) proposed the assessment of three parameters, including AUC, induction time, and FH decay rates, as shown in Figure 3A. Induction time reflects the quantity of antioxidant compounds that demonstrated radical scavenging activity, whereas the comparison of FH decay rates after induction time provides better visualization of the formation of by‐products upon radical scavenging by antioxidants, which carry on their protective effect. However, the induction time measurement was recommended for standard antioxidant and/or fast radical scavenging compounds, whereas for complex natural extracts, integrated analysis of AUC, induction time, and FH decay was recommended. This multi‐parametric approach accounts for the magnitude and duration of antioxidant activity, accommodates non‐linear dose–response relationships caused by synergistic or antagonistic interactions among components, and resolves overlapping kinetic phases (e.g., primary vs. secondary oxidation inhibition). Together, these metrics provide a holistic evaluation of total antioxidant capacity in heterogeneous matrices like plant extracts or food systems (Apak et al. 2016a).

Recently, Asma et al. (2024) offered a comprehensive ORAC kinetic model encompassing nine distinct reactions (Figure 3B). The kinetic model begins with the *initiation* reaction (Reaction 1), where AAPH undergoes thermal decomposition, producing nitrogen gas (N_2_) and alkyl radicals (R•). These radicals react with oxygen to form peroxyl radicals (ROO•), generated at a constant rate (*R_i_*) under controlled conditions (pH 7, 37°C). This model assumed that *R_i_* remained constant due to the excess AAPH present. Next, *peroxyl radical termination and alkoxyl radical formation* (Reactions 2 and 3) occurred. In the absence of antioxidants (AH), ROO• radicals self‐react to form an unstable tetroxide intermediate that decomposes into alkoxyl radicals (RO•) and oxygen (O_2_) (Asma et al. 2024). Most RO• radicals recombine to form non‐radical products (NRPs), whereas a minority escape to participate in further reactions (López‐Alarcón et al. 2020). The termination of ROO• radicals was assumed to occur at a rate constant (*k*_2_) of 1 × 10^6^ M^−1^ s^−1^, and that of RO• radicals at *k*_7_ = 1 × 10^9^ M^−1^ s^−1^. Reaction 4 involved *FH bleaching*, primarily mediated by RO• radicals, with negligible interaction with ROO• radicals (Asma et al. 2024). It was assumed that FH bleaching was mediated predominantly by RO• radicals (Dorta et al. 2015), with a minimum rate constant (*k*_4_) of 1 × 10^7^ M^−1^ s^−1^, as higher values did not affect bleaching kinetics (Asma et al. 2024). *AH activity* (Reaction 5) involved AH scavenging ROO• radicals through a proton‐coupled electron transfer mechanism, involving either HAT or electron transfer with subsequent protonation or deprotonation (Amorati et al. 2016). A *repair mechanism* (Reaction 6) allowed AH to regenerate FH by interacting with F•, governed by the equilibrium constant (*k*_6_), with higher values reflecting stronger electron‐donating capacities. Finally, *radical termination* (Reactions 7–9) reduced all the radical species, including RO•, A•, and F•, into stable NRPs. The self‐termination of F• and A• (Reactions 8 and 9) was assumed to occur at a rate constant (*k*_8_ *= k*_9_) of 1 × 10^8^ M^−1^ s^−1^ (Asma et al. 2024).

Moreover, it should be highlighted that to determine the AH reactivity rate constant (*k*_5_), and FH regeneration constant (*k*_6_), the values for *R_i_* and *k*_3_ were tuned using Trolox, whereas *k*_2_, *k*_4_, *k*_7_, *k*_8_, and *k*_9_ were fixed at the values described above.

This kinetic model, supported by reasonable assumptions, offers a robust framework for understanding the dynamics of radical interactions and the antioxidant capacity of different compounds.

##### 3.1.2.1 Applications of ORAC Kinetics

###### 3.1.2.1.1 Standard Antioxidants

The ORAC kinetic model reported by Asma et al. (2024) offers a robust framework for evaluating antioxidant activity by integrating parameters, such as reactivity rate constants, FH repair mechanisms, and stoichiometry. This kinetic approach surpassed conventional AUC‐based evaluations, providing a more precise understanding of the dynamics of radical scavenging and secondary antioxidant functions. A key metric derived from the kinetic model was the reactivity rate constant (*k*_5_), which quantifies the rate at which antioxidants neutralize free radicals. Among the tested standard antioxidants, Trolox demonstrated the highest reactivity (*k*_5_ = 4.0 × 10^5^ M^−1^ s^−1^) (Asma et al. 2024), consistent with its highly electron‐dense chromanol ring that enhances HAT and facilitates resonance stabilization of the resultant radical (Haidasz et al. 2016; Jodko‐Piórecka et al. 2022). Sinapic acid, with two ortho‐methoxyl groups, exhibited significant reactivity (*k*_5_ = 60 ± 2 × 10^3^ M^−1^ s^−1^), although markedly lower than Trolox (Asma et al. 2024). This highlights the critical role of methoxyl substitutions in stabilizing phenoxyl radicals (Charlton et al. 2023). In contrast, ferulic acid (*k*_5_ = 30 ± 2 × 10^3^ M^−1^ s^−1^; one methoxyl group) and *p*‐coumaric acid (*k*_5_ = 20 ± 2 × 10^3^ M^−1^ s^−1^; no methoxyl group), having less electron‐donating groups (Upadhyay and Mohan Rao 2013), exhibited lower radical‐scavenging rates. Furthermore, caffeic acid, featuring two hydroxyl groups and a conjugated double bond, and chlorogenic acid, which has an esterified quinic acid component, showed substantial radical‐scavenging activity, although this was slightly lower than that of sinapic acid (Table 2) (Asma et al. 2024). Notably, the kinetic‐based reactivity provided a different ranking compared to the AUC‐derived assessments, where *p*‐coumaric acid appeared stronger. This discrepancy underscores the limitations of AUC‐based methods in accurately capturing the dynamic interplay between reaction kinetics and antioxidant capacity, emphasizing the importance of rate constants for evaluating antioxidant effectiveness (Asma et al. 2024).

In addition to reactivity, the ORAC assay captured the repair mechanism of antioxidants through the equilibrium constant (*k*_6_), which quantified their ability to regenerate fluorescein (Asma et al. 2024). Again, Trolox exhibited the highest FH repair capability, attributed to its low redox potential (*E_p,a_* = +80 mV) (Selaković et al. 2023), significantly lower than fluorescein's redox potential (*E_p,a_* = +750 mV) (Queiroz et al. 2017). This aligns with its pronounced radical‐scavenging activity and highlights its dual functionality in the ORAC assay. Sinapic acid, with a moderate redox potential (*E_p,a_* = +188 mV) (Teixeira et al. 2013), also demonstrates notable fluorescein repair, followed by ferulic acid and *p*‐coumaric acid (Asma et al. 2024). Interestingly, *p*‐coumaric acid, despite its relatively weak radical‐scavenging activity, contributed to FH regeneration. This explains the extended fluorescein bleaching curves observed (Figure 3C), where its slower interaction with peroxyl radicals was offset by its repair capacity. The prolonged fluorescence signal, particularly for *p*‐coumaric acid, reflected a delicate balance between its persistence in solution and its ability to reduce oxidized fluorescein. This dual role further illustrates the complexity of antioxidant behavior within the ORAC framework, where both primary and secondary activities significantly influence the overall efficacy (Asma et al. 2024).

Additionally, the stoichiometry factor, representing the molar ratio of reactive species scavenged per antioxidant molecule, provides additional insights into antioxidant capacity. Monophenolic cinnamic acids, such as *p*‐coumaric acid and sinapic acid, exhibited stoichiometry values ranging from 1.0 to 2.0. In contrast, ortho‐diphenolic cinnamic acid, like caffeic acid, exhibited higher stoichiometry values (e.g., 4.0), reflecting its potential to generate additional active phenolic intermediates (Asma et al. 2024). This difference arises from the ortho‐diphenolic structure's ability to form stable quinones and subsequent reactive species (Saito et al. 2006). However, stoichiometry alone is insufficient to define antioxidant performance. For instance, although *p*‐coumaric acid and Trolox shared comparable stoichiometry values, their reactivity rates differed significantly, as evidenced by their respective *k*_5_ values. This divergence calls attention to the importance of integrating stoichiometric and kinetic parameters to accurately evaluate the antioxidant efficacy. Antioxidants with faster reaction rates are often more effective in mitigating oxidative damage, even when present in smaller quantities or exhibiting lower stoichiometry (Asma et al. 2024).

###### 3.1.2.1.2 Fruit Juices

Extending the application of the kinetic model, its performance was evaluated in real food systems, such as fruit juices. Remarkably, the kinetic model revealed a reactivity order (*k*_5_)—lemon ∼ mango > orange ∼ grape ∼ pomegranate > pineapple > apple—that varied significantly (*p* < 0.05) from traditional ORAC assay results. An appropriate example illustrating this divergence was the comparative analysis of lemon and apple juices. In traditional AUC‐based ORAC assays, apple juice exhibited higher AUC values than lemon juice, indicating superior antioxidant properties. However, this conflicts with empirical culinary practices, where lemon juice is widely employed for its antioxidative efficacy in food preservation. The kinetic model resolved this contradiction by demonstrating that lemon juice, despite its lower AUC, possessed the highest *k*_5_ value, reflecting rapid radical neutralization by fast‐acting antioxidants such as ascorbic acid. These compounds act swiftly, neutralizing radicals early in the reaction and thereby reducing the total AUC. In contrast, apple juice contains slower‐reacting antioxidants, which prolong radical scavenging, resulting in higher AUC values but lower *k*_5_. These results highlight the kinetic model's ability to reconcile practical observations with experimental results, providing a more accurate representation of antioxidant dynamics in food systems (Asma et al. 2024).

Collectively, these findings demonstrate the intricate mechanisms underlying the ORAC assay, where FH bleaching reflects not only direct radical scavenging but also the regenerative capacity of antioxidants. The kinetic model thus provides a robust tool for deciphering the multifaceted roles of antioxidants, facilitating a more refined and accurate assessment of their potential applications across various domains.

##### 3.1.2.2 Advantages and Limitations of ORAC Kinetics

The kinetic model presented by Asma et al. (2024) significantly improved the accuracy of antioxidant reactivity assessments in ORAC experiments. This model quantified reaction rates between antioxidants and radicals while accounting for the role of alkoxyl radicals in fluorescein bleaching and the dual functions of antioxidants in scavenging peroxyl radicals and regenerating oxidized fluorescein. Moreover, the application of the model to individual antioxidants and fruit extracts provided absolute reactivity rate constants, offering a more objective, reproducible, and transferable measure of the antioxidant activity compared to the traditional AUC‐based methods. By transitioning from conventional indices to a kinetic‐based approach, the model provides deeper insights into antioxidant behavior and establishes direct links between reactivity rate constants and molecular structures, identifying critical structural features influencing antioxidant interactions with fluorescein or other target molecules. This advancement addresses the structure–activity relationship gap highlighted by Schaich et al. (2015).

On the other side, the complexity of the ORAC reaction introduces numerous challenges for researchers, ranging from experimental setup to data interpretation, which can compromise the accuracy and reliability of the results and should therefore be carefully considered. First, precise temperature control is critical for consistent radical generation and reaction completion, but temperature variations, especially in plate readers, can lead to slow or incomplete reactions, which can falsely increase the value of the antioxidant activity. Oxygen availability is another key factor. Insufficient oxygen, due to prolonged heating or pre‐warming, can result in inconsistent reaction rates and unreliable rate constant estimation (Schaich et al. 2015). Reagent concentrations, particularly FH and AAPH, must be carefully balanced. High FH levels can cause quenching, whereas inadequate AAPH concentrations hinder reaction completion, distorting kinetic profiles. Furthermore, the evaporation of organic solvents during long kinetic studies can alter reagent concentrations and introduce errors in the reaction kinetics. Non‐standardized phenol content in extracts introduces variability, and non‐radical interactions, such as fluorescence quenching or stabilization, further skew kinetic data. Long reaction times intensify these issues by increasing the appearance of side reactions, oxygen depletion, and solvent evaporation, leading to over/underestimation of antioxidant activity.

In addition to experimental challenges, data analysis involves setting up models that account for multiple reactions and rate constants, requiring a strong grasp of biochemical processes to accurately define reaction mechanisms. Parameter estimation can be computationally challenging due to the need to optimize numerous variables simultaneously, leading to convergence issues or inconsistencies in derived rate constants. Moreover, the modeling process often relies on assumptions about reaction rates and mechanisms, which may not always align with actual experimental conditions, potentially limiting the model's accuracy and broader applicability.

Overall, these challenges highlight the need for rigorous experimental control, systematic optimization, and protocol standardization to ensure reliable data for kinetic modeling and rate constant determination.

#### 3.1.3 Oxidizable Substrate Monitoring

The ability of antioxidants to inhibit oxidative degradation in food products is commonly evaluated by analyzing the formation of oxidation products. Traditional methods include the quantification of hydroperoxides via iodometric titration or colorimetric assays, which involve the oxidation of Fe^2^⁺ to Fe^3^⁺ and subsequent formation of colored iron complexes. Conjugated dienes are typically measured using UV–Vis spectrophotometry, whereas volatile oxidation products such as hexanal are detected through gas chromatography–mass spectrometry (Amorati and Valgimigli 2018; Thomsen et al. 2016). Despite their utility, these approaches are prone to interference and rely on discontinuous sampling, which may inaccurately reflect the effectiveness of antioxidants in mitigating lipid oxidation. Moreover, modeling the antioxidant activity in polar organic solvents is comparatively straightforward, whereas replicating these conditions in complex food systems is significantly more challenging.

To overcome these limitations, the use of lipid‐soluble fluorescent molecular probes offers a practical solution for real‐time monitoring of the autoxidation process (Amorati and Valgimigli 2018; Laguerre et al. 2007). C11‐BODIPY^581/591^ has been extensively employed to track lipid oxidation (Kusio and Litwinienko 2022) due to its susceptibility to oxidation in azo‐initiated co‐oxidation reactions with methyl linoleate, and the addition of the antioxidant BHT (2,6‐di‐*tert*‐butyl‐4‐methylphenol) has been shown to inhibit this oxidation process (Yoshida et al. 2003). On the basis of this foundation, Pratt et al. (Haidasz et al. 2016; Shah et al. 2019) developed a modified, less oxidizable version of C11‐BODIPY^581/591^, referred to as styrene‐conjugated BODIPY (STY‐BODIPY). This probe enabled antioxidant kinetic studies of peroxidation in systems like styrene, cumene, and tetrahydrofuran.

**FIGURE 4:**
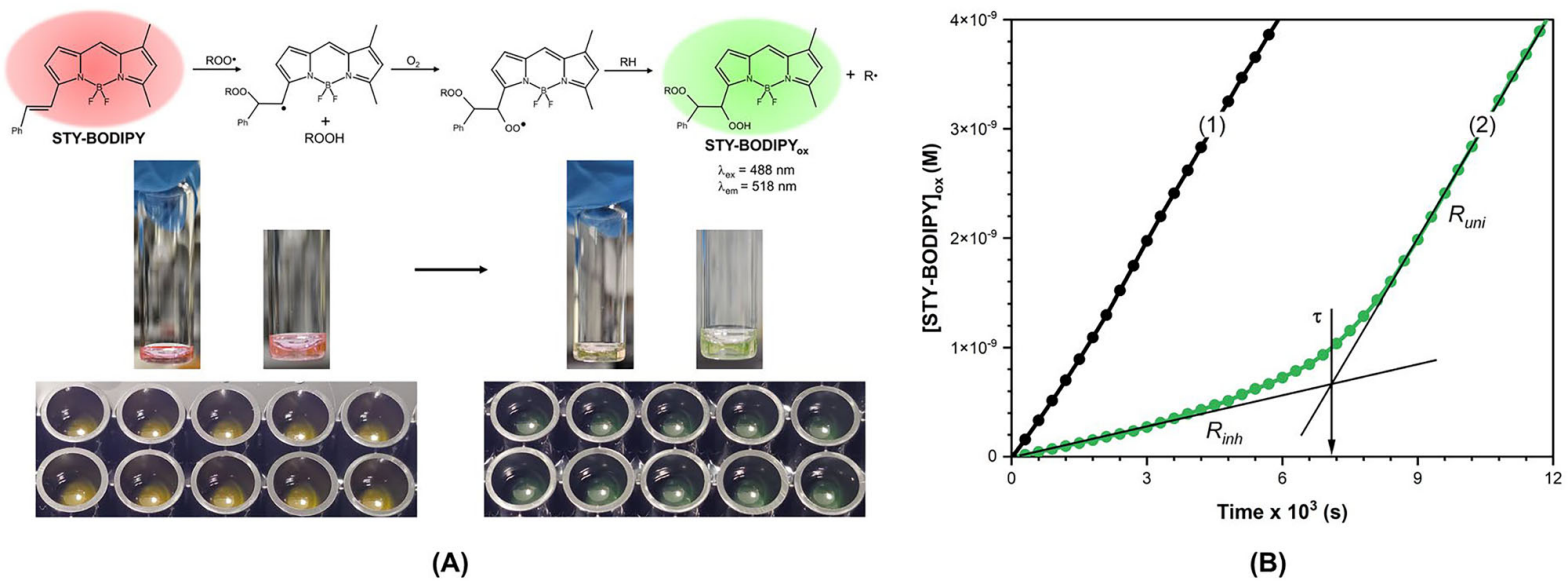
(A) Reaction scheme of STY‐BODIPY with peroxyl radicals to form the oxidized form (STY‐BODIPY_ox_) used as a signal carrier in co‐autoxidation with stripped sunflower oil, accompanied by a visual depiction of the color transformation of STY‐BODIPY to STY‐BODIPY_ox_, shown in a transparent vial and a 96‐well plate, and (B) STY‐BODIPY_ox_ formation with time initiated by AIBN (50 mM) with no added antioxidant (Trace 1) and inhibited by PMC antioxidant (Trace 2) at 30°C. Key parameters: *R*_inh_ (rate of inhibited co‐autoxidation), *R*_uni_ (rate of uninhibited co‐autoxidation), and *τ* (induction time). *Source*: Reprinted from Suhag, Jin, et al. (2024) under a Creative Commons Attribution 4.0 International License.

Expanding on these advancements, Suhag, Jin, et al. (2024) introduced a continuous fluorescence‐based assay for antioxidant testing, using stripped sunflower oil (oil depleted of natural antioxidants) as the oxidizable substrate. In its reduced form, STY‐BODIPY emits a pseudo‐red fluorescence. Upon interaction with chain‐propagating peroxyl radicals (ROO•), the styryl moiety is oxidized, forming non‐conjugated products, which shift the fluorescence emission from 565 to 518 nm, resulting in a green fluorescence (Figure 4A). The oxidation process was tracked by monitoring the fluorescent oxidized product (STY‐BODIPY_ox_, *λ*_ex_ = 488 nm, *λ*_em_ = 518 nm). Measuring the rate of STY‐BODIPY_ox_ formation in the presence of an antioxidant allowed the determination of the antioxidant reactivity in inhibiting the co‐autoxidation of stripped sunflower oil, as calculated using Equation (3) (Shah et al. 2019; Suhag, Jin, et al. 2024):

(3) $$R_{\mathrm{inh}}=\frac{\delta {\left[\mathrm{STY}-\mathrm{BODIPY}\right]}_{\mathrm{ox}}}{\delta t}=\frac{k_{\mathrm{ST}}\left[\mathrm{STY}-\mathrm{BODIPY}\right]R_{i}}{nk_{\mathrm{inh}}\left[\mathrm{AH}\right]}$$

(4) $$R_{i}=\frac{n\;[\mathrm{AH}]}{\tau }$$

where *k*_ST_ represents the rate constant for the reaction between the peroxyl radical (ROO•) and STY‐BODIPY, whereas *k*_inh_ refers to the inhibition rate constant, reflecting the reactivity of the antioxidant, [AH] is the molar concentration of the antioxidant, and *R_i_* is the rate of initiating radical formation due to the thermal decomposition of the radical initiator (AIBN). The value of *R_i_* was determined using Equation (4) in the presence of a known concentration of an antioxidant with a defined stoichiometry (*n*). It is worth mentioning that the concentration of the radical initiator is generally used in millimolar range, and the amount of initiator consumed during the reaction is very small, allowing *R_i_* to be assumed as a constant along the reaction (Suhag, Ferrentino, et al. 2024).

During AIBN‐initiated autoxidation, STY‐BODIPY underwent co‐autoxidation with sunflower oil, as demonstrated by the gradual increase in the concentration of STY‐BODIPY_ox_ over time (Figure 4B), indicating a continuous oxidation process. The presence of an antioxidant decelerated this process, reducing the rate of STY‐BODIPY_ox_ formation and creating a phase of delayed oxidation referred to as the inhibition period (Shah et al. 2019; Suhag, Jin, et al. 2024). The rate observed during this phase, known as the inhibition rate (*R*_inh_), reflects the antioxidant's effectiveness in slowing down the oxidation. The duration of this phase, defined as the induction time (*τ*) as the point at which all active antioxidants in the sample have been consumed by radicals. The induction time is a crucial parameter for evaluating the antioxidant performance, with longer *τ* values signifying higher antioxidant capacity (Y. Guo, Baschieri, et al. 2021; Suhag, Razem, et al. 2024). These parameters were instrumental in determining the antioxidant inhibition rate constant (*k*_inh_).

##### 3.1.3.1 Applications of Oxidizable Substrate Monitoring

Suhag, Jin, et al. (2024) examined the synergistic interactions between 2,2,5,7,8‐pentamethyl‐6‐chromanol (PMC, an analog of α‐tocopherol) and various antioxidants in inhibiting sunflower oil oxidation. Among non‐phenolic compounds, γ‐terpinene—a pre‐aromatic terpene commonly found in essential oils—exhibited limited activity when used alone, even at millimolar concentrations. It was able to just slow down the co‐autoxidation of STY‐BODIPY in sunflower oil without producing a clear induction period. However, when combined with PMC, γ‐terpinene significantly increased the induction time compared to PMC alone. Furthermore, the induction time showed a linear increase with concentrations of γ‐terpinene in the presence of PMC. Although the inhibition rate constant (*k*_inh_) for the γ‐terpinene and PMC combination was similar to that of PMC alone, 1.5 × 10^6^ M^−1^ s^−1^ (Baschieri et al. 2019), the stoichiometry value (*n*) was substantially greater than 2 (Table 2) (Suhag, Jin, et al. 2024). This synergistic interaction is attributed to the oxidation of γ‐terpinene, which generates hydroperoxyl radicals (HOO•) capable of both reducing and oxidizing. These radicals can donate hydrogen atoms to PMC radicals, regenerating the active phenolic form of PMC (Foti and Ingold 2003; Y. Guo, Baschieri, et al. 2021).

Moreover, quercetin and caffeic acid demonstrated synergistic effects with PMC, extending the induction time in a concentration‐dependent manner (Suhag, Jin, et al. 2024). The synergistic mechanism in these cases involves a regeneration process. PMC, acting as a chain‐breaking antioxidant, donates hydrogen atoms to alkyl peroxyl radicals, forming PMC radicals. Quercetin and caffeic acid then donate hydrogen atoms to the PMC radicals, restoring active PMC and forming quercetin quinone (Y. Zhang, Wang, et al. 2023). Notably, γ‐terpinene required higher concentrations (millimolar) to achieve synergy compared to the micromolar levels effective for quercetin and caffeic acid. This reflects distinct underlying mechanisms (Y. Guo, Baschieri, et al. 2021; Suhag, Jin, et al. 2024).

##### 3.1.3.2 Advantages and Limitations of Oxidizable Substrate Monitoring

This method offered a notable advantage by enabling the study of antioxidant reactivity kinetics in a food‐based oxidizable substrate using straightforward and widely accessible laboratory equipment. The incorporation of a microplate system allowed for the simultaneous analysis of multiple samples, providing a high‐throughput capability. This setup reduced variability and ensured greater consistency by processing all samples under uniform conditions. It is especially beneficial for kinetic studies, as real‐time monitoring across multiple samples enhances accuracy and precision. Furthermore, the low sample volume requirements per well make it economical and efficient compared to single‐cell fluorimeters, which typically require larger sample volumes and handle samples individually. For laboratories without access to microplate‐based systems, single‐sample fluorimeters with temperature control can still be utilized effectively, though with lower throughput and higher manual effort (Suhag, Jin, et al. 2024).

Although the fluorescent probe method offers many advantages, several limitations must be considered. Although commercially available, these probes are expensive. They can be synthesized in a laboratory setting, but this requires advanced expertise in chemistry, which may not be accessible to all researchers. Additionally, despite their potential, fluorescent probe‐based approaches have not been extensively explored and require further validation across diverse food‐based substrates.

Applying this method to complex food matrices, such as emulsions, presents significant challenges due to fluorescence interference caused by the heterogeneous nature of these systems. Components like lipids, proteins, and surfactants can contribute to light scattering and autofluorescence, which may overlap with the emission spectra of fluorescent probes (e.g., STY‐BODIPY), complicating signal acquisition and reducing measurement accuracy. For example, lipid droplets in emulsions scatter light, distorting fluorescence intensity, whereas phenolic compounds or chlorophyll residues in plant‐based extracts may autofluoresce at wavelengths overlapping with the probe emission range. These interferences lead to increased background noise, hiding the true signal of the target oxidizable substrates. Furthermore, fluorescent probes may participate in side reactions during oxidation studies, which could lead to an underestimation of antioxidant reactivity. Addressing these limitations will require continued research and optimization to fully acquire the potential of this method.

### 3.2 Oxygen Uptake Method

The unifying aspect of oxidation across various substrates is oxygen consumption. Oxidation results from the reaction of dissolved or headspace oxygen with an initiating alkyl radical at a diffusion‐controlled rate (*k_o_* ∼ 10^9^ M^−1^ s^−1^), generating a peroxyl radical (Johnson and Decker 2015). The reaction occurs rapidly because there are no significant thermodynamic, kinetic, or quantum mechanical barriers between the unpaired electrons of oxygen and the alkyl radical (Dunford 1987). Subsequently, during the propagation phase, each peroxyl radical abstracts a hydrogen atom from lipid molecule, producing a lipid hydroperoxide and regenerating an alkyl radical. This cycle consumes additional oxygen molecules, sustaining the radical chain reaction. Consequently, oxygen consumption serves as both a direct and quantitative measure of oxidation progression, as each propagation step stoichiometrically consumes oxygen (Durand et al. 2025; Johnson and Decker 2015). Oximetry setup offers a highly versatile method for studying this process in diverse samples. Oximetry methods determine oxygen levels in a closed system containing an oxidizable substrate undergoing oxidation. Oxygen levels can be measured through two primary approaches.

**FIGURE 5:**
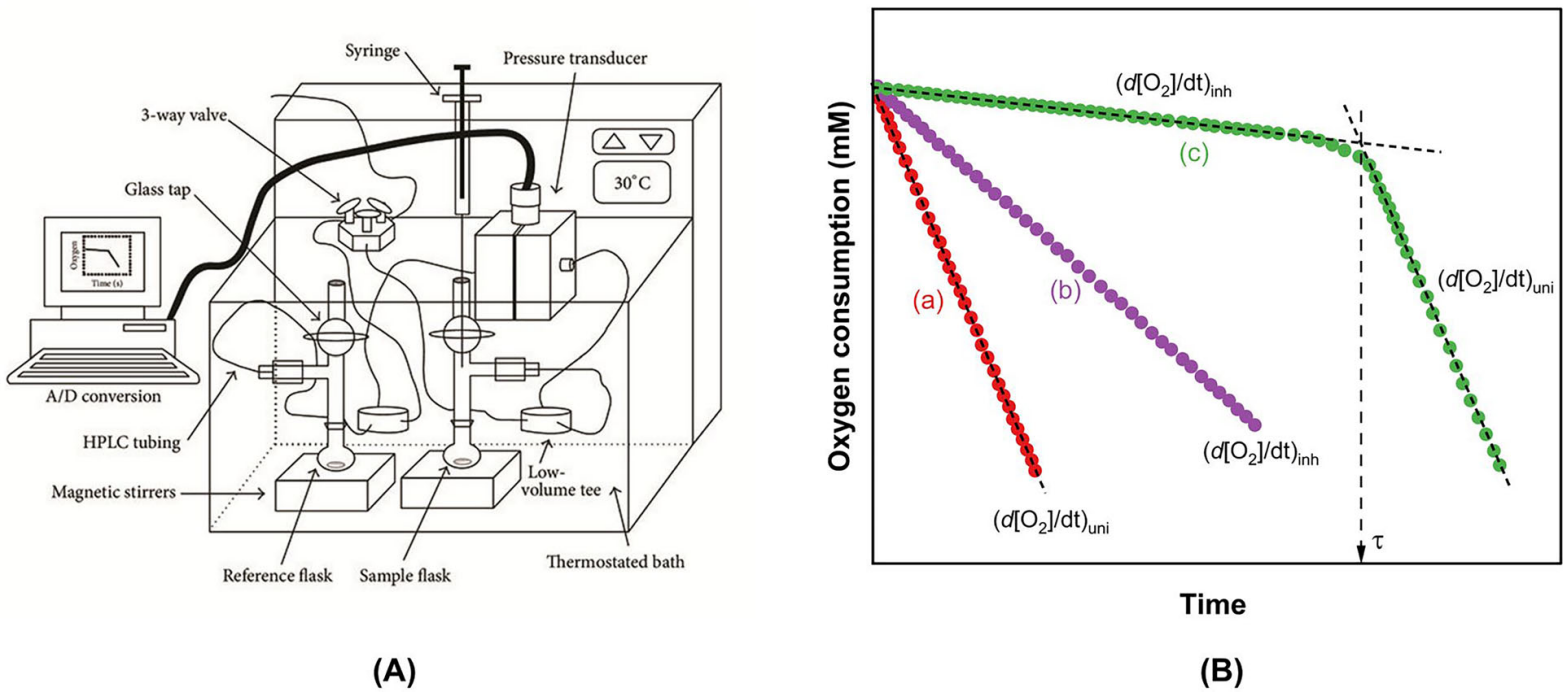
(A) Differential oxygen uptake apparatus for autoxidation kinetics and (B) schematic representation of oxygen consumption versus time plots during the autoxidation of a standard substrate added with (a) no antioxidant, (b) a modest chain‐breaking antioxidant, and (c) good chain‐breaking antioxidant. HPLC, high‐performance liquid chromatography. *Source*: (A) Reprinted from Valgimigli and Pratt (2012) with permission from John Wiley and Sons.

***Pressure‐Based Measurement***: Oxygen consumption is inferred from the pressure drop in a closed system, which can be measured using various pressure gauges. Differential pressure transducers, for instance, detect small pressure differences between a sample flask and a reference flask (Figure 5A). These transducers are suitable for both organic solvents and aqueous solutions. The reference flask contains the same reaction mixture as the sample but includes a high concentration of an antioxidant, which compensates for pressure reductions caused by nitrogen formation from the azo‐initiator and oxygen depletion during substrate autoxidation (Amorati et al. 2017; Burton and Ingold 1981). A detailed discussion on the differential pressure oximetry setup, experimental procedure, calibration, and data analysis can be found in Amorati et al. (2017).

***Direct Oxygen Measurement***: Oxygen concentration can be directly measured either in the solution or in the headspace of a closed vial using oxygen‐sensitive probes. Optical and electrochemical sensors are the primary tools for this method. The optical technique involves fluorescence quenching by oxygen, where a fluorescent probe attached to the tip of an optical fiber is positioned in the sample or headspace. This approach is effective in both air and water but is incompatible with organic solvents (Guernelli et al. 2020; Mollica et al. 2020). The electrochemical method employs a polarographic Clark electrode, which detects oxygen by generating a current proportional to the oxygen diffusing to the electrode tip through a polymer membrane. This method is suitable for aqueous systems (Grebowski et al. 2020).

During autoxidation experiments in the presence of antioxidants, oxygen consumption typically follows a biphasic pattern. Initially, there is a period of slow oxygen consumption, known as the inhibited phase with a specific rate (*R*_inh_), during which the antioxidant suppresses autoxidation. This is followed by a rapid increase in oxygen consumption during the uninhibited phase with a specific rate (*R*_uni_), as shown in Figure 5B. The rate of initiation (*R_i_*) is crucial for calculating key antioxidant parameters, and *R_i_* cannot be determined directly. *R_i_* is determined indirectly (Equation 4) by measuring the length of inhibited peroxidation (i.e., the induction period, *τ*) in the presence of an antioxidant with a known concentration and stoichiometric factor (*n*) (Bravo‐Díaz 2023).

The duration of the inhibited phase (*τ*) reflects the antioxidant's stoichiometry, whereas comparing the oxygen uptake rates in the inhibited (*R*_inh_) and uninhibited (*R*_uni_) phases provides the rate constant for the reaction of antioxidants with peroxyl radicals (*k*_inh_) using Equations (5) and (6) (Baschieri et al. 2019; Baschieri and Amorati 2021).

(5) $$R_{\mathrm{uni}}=-{\left(\frac{d[O_{2}]}{\mathrm{dt}}\right)}_{\mathrm{uni}}=\frac{k_{p}}{\sqrt{2k_{t}}}\times [\mathrm{RH}]\times \sqrt{R_{i}}\;$$

(6) $$R_{\mathrm{inh}}=-{\left(\frac{d[O_{2}]}{\mathrm{dt}}\right)}_{\mathrm{inh}}=\frac{k_{p}}{k_{\mathrm{inh}}}\;\times \frac{[\mathrm{RH}]\times R_{i}}{n\times \left[\mathrm{AH}\right]}$$

where [O_2_] and [RH] represent the molar concentration, respectively, of oxygen and lipid substrate; *k_p_*, *k_t_*, and *k*_inh_ are the rate constants of the propagation, termination, and inhibition reactions, respectively (Scheme 1).

#### 3.2.1 Applications of Oxygen Uptake Method

##### 3.2.1.1 Essential Oils

Guo, Pizzol, et al. (2021) used the oxygen uptake method to assess the antioxidant reactivity of five phenol‐rich essential oils. Among the tested oils, the terpene‐rich *Thymus vulgaris*, *Origanum vulgare*, and *Satureja hortensis* exhibited similar inhibition constant values, on the order of 10^4^ M^−1^ s^−1^ at 30°C. In contrast, the phenylpropanoid‐rich *Eugenia caryophyllus* and *Cinnamomum zeylanicum* displayed inhibition constants in the range of 10^3^ M^−1^ s^−1^. All essential oils provided strong antioxidant protection for cumene at a concentration of 1 ppm (1 mg L^−1^). Additionally, they offered excellent protection for squalene when their concentration was adjusted to 100 mg L^−1^ to compensate for the higher oxidizability of this substrate.

A recent study by Pan et al. (2024) from the same research group further explored 13 essential oils from 11 botanical species. The essential oils of *Juniperus oxycedrus*, *Syzygium aromaticum*, *T. vulgaris*, *Thymbra capitata*, *Betula alba*, *Pimenta racemosa*, and *Satureja montana*, which contained 23%–86% phenolic components, exhibited inhibition constants in the order of 10^4^ M^−1^ s^−1^ at 30°C in cumene, comparable to the reference antioxidant BHT. However, the essential oils of *Apium graveolens*, *Daucus carota*, *Tagetes minuta*, and *Cedrus atlantica* did not show chain‐breaking activity. Furthermore, when tested for their ability to protect olive oil, the relative antioxidant efficacy differed from that observed in cumene. In olive oil, the ranking of antioxidant effectiveness was not the same as that in cumene and was as follows: *J. oxycedrus* > *B. alba* > *S. Montana* > *S. aromaticum* ≥ *T. vulgaris* ≈ *T. capitata* ≈ *P. racemosa* (Pan et al. 2024). The shift in the relative ranking was attributed to medium effects on the antioxidant kinetics of the phenolic components. Because triglycerides are strong hydrogen‐bond acceptors, they can reduce the antioxidant performance of strong hydrogen‐bond donor phenols due to the formation of intermolecular hydrogen bonds (Y. Guo, Pina, et al. 2024).

##### 3.2.1.2 Natural Antioxidants

Amorati et al. (2015) reported the reactivity constants of magnolol and honokiol, two bisphenolic neolignans found in the bark of *Magnolia officinalis*. In inhibited autoxidation experiments conducted at 30°C, magnolol was found to trap four peroxyl radicals, with an inhibition constant of 6.1 × 10^4^ M^−1^ s^−1^ in chlorobenzene and 6.0 × 10^3^ M^−1^ s^−1^ in acetonitrile. Honokiol, on the other hand, trapped two peroxyl radicals in chlorobenzene (*k*_inh_ = 3.8 × 10^4^ M^−1^ s^−1^) and four peroxyl radicals in acetonitrile (*k*_inh_ = 9.5 × 10^3^ M^−1^ s^−1^). The differences in their reactivity can be attributed to the presence of intramolecular hydrogen bonding, which occurs among the reactive hydroxyl (OH) groups in magnolol, as well as interactions between the OH groups and the aromatic and allyl π‐system.

In another study, nine acylated anthocyanin molecules were extracted from sprout juice. Their antioxidant potential was evident in aqueous micelles, where they consistently inhibited linoleic acid autoxidation, with an inhibition constant of 3.8 ± 0.7 × 10^4^ M^−1^ s^−1^ at 37°C. In acetonitrile, the inhibition constant varied on the basis of the degree of acylation, ranging from (0.9 to 2.1) × 10^5^ M^−1^ s^−1^ at 30°C. Each anthocyanin molecule neutralized between four and seven peroxyl radicals, with sinapic acid–acylated anthocyanins exhibiting stronger activity than those acylated with ferulic acid. Under identical conditions, deacylated cyanin, ferulic acid, and sinapic acid had inhibition constant values of 0.4 × 10^5^, 0.3 × 10^5^, and 1.6 × 10^5^ M^−1^ s^−1^, respectively, with trapping two to three peroxyl radicals (Matera et al. 2015).

#### 3.2.2 Advantages and Limitations of Oxygen Uptake Method

Oxygen uptake methods offer a reliable way to assess antioxidant reactivity by measuring oxygen consumption during inhibited autoxidation. This method provides valuable kinetic data, such as inhibition rate constants and reaction stoichiometry, allowing for a mechanistic understanding of antioxidant performance. The versatility of oxygen uptake methods also makes them applicable to a wide range of systems, including bulk oils, emulsions, micelles, and other lipid‐based matrices. Researchers can select different oxidizable substrates—such as cumene, linoleic acid, or squalene—to test antioxidants under conditions relevant to food and pharmaceutical applications. This adaptability allows for a more accurate assessment of antioxidant reactivity in real‐world scenarios.

However, these methods also have some limitations. Autoxidation reactions are inherently slow, making oxygen uptake methods more time‐consuming than techniques based on stable radical quenching. Additionally, measuring oxygen consumption requires specialized instrumentation, which is not widely available in many laboratories, even though it is not prohibitively expensive. Some equipment, such as pressure transducers used in the oxygen uptake studies, is not commercially available and must be assembled from individual components. Another drawback is that only one sample can typically be analyzed at a time, making the process labor‐intensive and limiting throughput (Amorati and Valgimigli 2015; Baschieri and Amorati 2021). These factors have restricted the widespread adoption of oximetry methods despite their advantages.

### 3.3 Calorimetry‐Based Methods

#### 3.3.1 Isothermal Calorimetry

**FIGURE 6:**
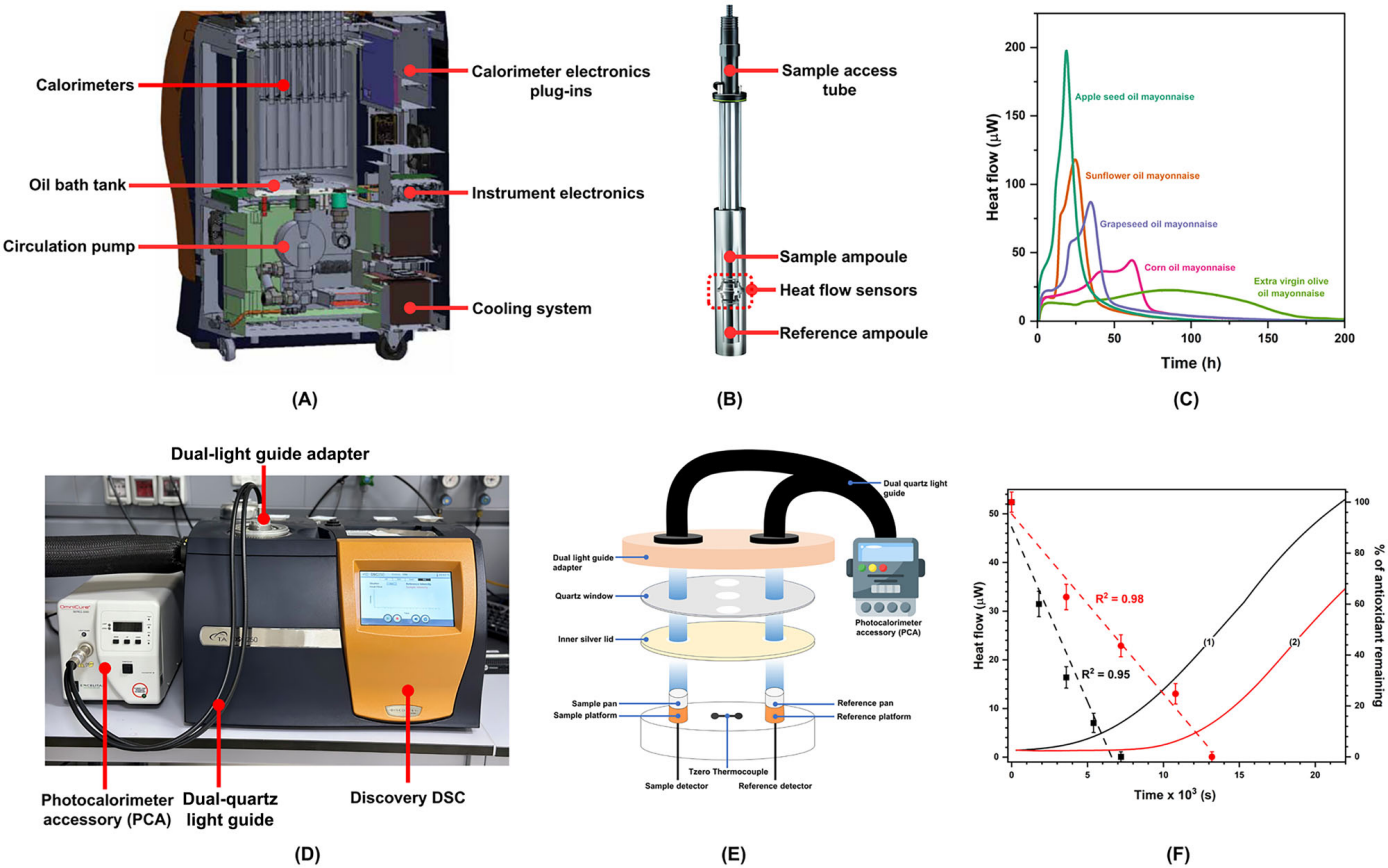
(A) Thermal activity monitor (TAM) and (B) calorimeter with sample ampoule, reference ampoule, and heat flow sensors, reprinted from TAM IV brochure, https://broch‐tam‐lv.pdf/20(tainstruments.com); (C) isothermal calorimetry trace of mayonnaise prepared using different oils undergoing oxidation at 60°C. (D) Photocalorimetry accessory (PCA) combined with differential scanning calorimetry (DSC) using a dual light guide, (E) schematic representation of the differential photocalorimetry setup, and (F) scatter points show the % of ferulic acid (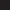) and caffeic acid (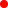) remaining during the photo‐oxidation of stripped linseed oil added with AIBN (5 mM) at 30°C; solid lines show the heat flow of stripped linseed oil added with (1) ferulic acid and (2) caffeic acid. *Source*: (C) Adapted from Suhag, Ferrentino, et al. (2024) under a Creative Commons license; (D), (E), and (F) reprinted from Suhag, Razem, et al. (2024) under a Creative Commons license.

Calorimetry, the study of heat measurement, presents an alternative means to quantify changes in processes and follow kinetics. Isothermal calorimeters employ a twin configuration, implying that the sample is positioned with a fixed reference in a vertical array with central heat flow detectors and heat sink located in a constant‐temperature liquid bath thermostat (Hofelich et al. 2001). The heart of the instrument is the liquid bath thermostat responsible for precise temperature control (Figure 6A). To conduct a calorimetric test, a small amount of sample (usually between 100 and 200 mg) is placed inside a glass ampoule of 4 mL capacity. This ampoule is then secured in a holder near a sensor that detects heat flow (Figure 6B). When the sample undergoes a reaction, either releasing or absorbing heat, its temperature changes, leading to a heat flow detected by the sensor as an electric voltage. The sensor operates based on the Seebeck principle, which states that a temperature variance across the sensor generates an electric voltage (Wadsö 2010). The voltage difference between the sample and reference sensors is measured, translated into thermal power over time, and then integrated to determine the total heat generated during the reaction. Thermal power indicates the reaction rate, whereas the total heat is proportional to the extent of the reaction (Hansen 2000; Willson et al. 1995). This utility stems from the correlation between the measured thermal power, denoted as *P* (*W*), and the rate of the process under examination, represented by *ν* (g s^−1^) (Equation 7). Additionally, the heat generated, denoted as *Q* (*J*) and quantified as the integrated thermal power, corresponds to the extent of the process advancement. This advancement is indicated by the mass, *m* (g), of the sample (or a portion thereof) that has undergone the reaction, which can also be expressed in molar quantities (Equation 8). Both these relationships are governed by the enthalpy change, represented as Δ*H* (J g^−1^), serving as the proportionality constant (Wadsö and Gómez Galindo 2009):

(7) P=ΔHv

(8) $$Q=\Delta H.\left(m_{0}-m\right)$$

Equations (7) and (8) provide the link between the calorimetric measurements (*P* and *Q*) and the corresponding kinetic rate law (*v* and *m*). In more intricate systems like food products and other complex materials, defining enthalpy change and rates presents challenges compared to simpler reactions. However, the heat measured can still be effectively used to model kinetics (Hansen 2000; Wadsö and Gómez Galindo 2009).

This flexibility lies in the consistent association of heat with any form of change. This universality enables calorimetry to detect and potentially measure alterations in a wide array of materials. The only requirements for the sample are that the process to monitor should generate a detectable amount of heat and that the sample, or at least a representative portion of it, fits within the calorimetric ampoule (O'Neill and Gaisford 2011).

Figure 6C shows calorimetric traces illustrating the process of autoxidation in mayonnaise prepared using different oils. At the start, there is minimal heat flow, indicating that autoxidation in the mayonnaise is inhibited by natural antioxidants present in the oil and is referred to as the “inhibited period.” However, after some time, a sharp increase in exothermic heat marks the beginning of lipid autoxidation. This time is referred to as the induction time (*τ*). Its occurrence clearly marks the time when all the antioxidants in the sample have been consumed by radicals. After the induction period, the oxidation process occurs uninhibited, referred to as the “uninhibited period.” Subsequently, the heat flow signal peaked and gradually decreased to nearly zero, signaling the completion of the autoxidation process. It is important to highlight that although every reaction of lipid peroxidation influences the heat measured by isothermal calorimetry, propagation reactions (Scheme 1) have a significant impact. This is because of their cyclical nature and increased substrate conversion for each radical generated by a radical initiator. Although the reaction of antioxidants with ROO• (inhibition reaction, Scheme 1) can generate heat, the minimal heat flow observed at the beginning of the reaction can be attributed to the antioxidants inhibiting propagation, thereby preventing the initiation of multiple cycles of the radical chain (Suhag, Ferrentino, et al. 2024).

For kinetic data analysis, the heat flow measured during calorimetric experiments is typically converted to oxygen consumption. The steps involved in this transformation, along with detailed kinetic modeling, have been discussed elsewhere (Suhag et al. 2025).

##### 3.3.1.1 Application of Isothermal Calorimetry

Suhag, Ferrentino, et al. (2024) examined the reactivity of natural antioxidants in different seed oils for inhibiting AIBN‐initiated mayonnaise oxidation using isothermal calorimetry at 60°C. The results showed that mayonnaise prepared with sunflower oil exhibited the highest inhibition constant, followed by apple seed > grapeseed > corn > extra virgin olive oil (Table 2). The superior reactivity of sunflower oil mayonnaise was attributed to the presence of lipid‐soluble antioxidants, such as tocopherols, which could rapidly neutralize peroxyl radicals. In contrast, extra virgin olive oil, which is particularly rich in polar phenolics like oleuropein and hydroxytyrosol (Frankel 2010), was less effective, as these compounds primarily function in the aqueous phase, making them less available to react quickly with peroxyl radicals formed in the lipid phase.

##### 3.3.1.2 Advantages and Limitations of Isothermal Calorimetry

Isothermal calorimetry is a powerful technique for studying antioxidant activity and oxidation kinetics, providing real‐time, continuous monitoring of heat flow associated with oxidation reactions. Unlike probe‐based methods, it requires minimal sample preparation and can be applied to various food matrices, including oils and emulsions. A major advantage is its ability to analyze up to 48 samples simultaneously, making it highly efficient for high‐throughput studies. Additionally, it allows direct measurement of reaction rates and kinetic parameters, offering valuable insights into antioxidant performance under realistic conditions (Suhag et al. 2025).

Despite its advantages, isothermal calorimetry has some limitations. The technique requires specialized and costly instrumentation, limiting its accessibility. It is also relatively slow compared to spectrophotometric methods, as oxidation reactions can take hours or even days to complete. A notable characteristic of isothermal calorimetry is its non‐specific heat flow measurement, which can be both advantageous and challenging. On one hand, this allows the technique to investigate samples with diverse physical and chemical properties, making it versatile. On the other hand, the recorded thermal signals may reflect not only lipid oxidation reactions but also contributions from physical processes (e.g., moisture loss and lipid phase changes), chemical side reactions (e.g., Maillard browning), and biological activity (e.g., microbial spoilage). These interferences can be minimized through careful experimental design and validation. For example, conducting calorimetric runs at typical storage temperatures (25–50°C) reduces heat signals from many physical and chemical transitions, and adding an antimicrobial agent (commonly sodium azide) suppresses microbial activity. Furthermore, complementary techniques such as differential scanning calorimetry (DSC) or thermogravimetric analysis (TGA) can be used to characterize sample thermal stability or track mass loss, helping to identify the most appropriate experimental conditions. Additionally, the interpretation of calorimetric data often requires advanced kinetic modeling, adding complexity to the analysis (Suhag et al. 2025).

#### 3.3.2 Differential Photocalorimetry

The DSC technique is a versatile tool for evaluating oil oxidative stability in both isothermal and non‐isothermal modes. The isothermal approach measures the oxidation induction time at a constant temperature, assessing oil resistance to oxidation. In contrast, the non‐isothermal mode involves heating the oil at a set rate to determine the oxidation onset temperature (Islam et al. 2022; Siejak et al. 2025). To utilize the potential of DSC technique and address challenges such as intermittent testing and the “memory effect” associated with periodic sampling from a single container in photo‐oxidation studies, we recently introduced the differential photocalorimetry (DPC) method. This method integrates a standard DSC unit with a UV–Vis light source using a dual‐quartz light guide (Figure 6D) (Suhag, Razem, et al. 2024). This arrangement enabled real‐time monitoring and enhanced the accuracy of kinetic data in photo‐oxidation studies.

In this setup, the light source provides continuous exposure to the sample under well‐controlled conditions, whereas the DSC continuously measures the heat flow associated with the photochemical reactions occurring in the sample. Figure 6E presents a schematic representation of the setup and its components. The dual‐quartz light guide ensures uniform and continuous light exposure to both the sample and the reference pan, effectively eliminating heat flow artifacts due to direct light exposure. This arrangement offers improved control over experimental conditions, such as temperature and airflow, using the DSC unit. Additionally, the light source, a 200 W high‐pressure mercury vapor short‐arc lamp, provides a broad range of light intensities and wavelengths, further enhancing the method's versatility (Suhag, Razem, et al. 2024).

##### 3.3.2.1 Applications of DPC

The DPC method was validated by assessing the role of antioxidants in inhibiting the photo‐oxidation of stripped linseed oil. For this purpose, ferulic acid was used as a reference antioxidant, effectively inhibiting the photo‐oxidation reaction in the oil and increasing the induction time in a concentration‐dependent manner. The kinetic analysis allowed determining the inhibition constant of ferulic acid to be 7.2 × 10^4^ M^−1^ s^−1^, with a stoichiometry (*n*) of 1. Among the tested antioxidants, caffeic acid, PMC, and sinapic acid exhibited the highest reactivity, followed by ferulic acid > *p*‐coumaric acid. Additionally, the depletion of antioxidants during the photo‐oxidation process was monitored using high‐performance liquid chromatography with a diode‐array detector (HPLC‐DAD). The complete consumption of antioxidants measured by HPLC‐DAD aligned with the induction time (*τ*) determined by DPC (Figure 6F), confirming the method's applicability for tracking photo‐oxidation kinetics. Finally, the method was applied to four commercially available vegetable oils, and the results were compared with those obtained from isothermal calorimetry (performed in the dark). Interestingly, both methods provided the same ranking of oils in terms of oxidative stability. However, the experiments under light exposure were completed 32 times faster, demonstrating the significant acceleration of the oxidation process by light (Suhag, Razem, et al. 2024).

##### 3.3.2.2 Advantages and Limitations of DPC

DPC offers several advantages for studying photo‐oxidation kinetics. A key benefit is its ability to provide real‐time and continuous monitoring of heat flow associated with photochemical reactions, eliminating the need for intermittent sampling. The recorded heat flow over time enabled the determination of key kinetic parameters. Additionally, the ability to precisely adjust light intensity makes DPC a valuable tool for routine screening of oil stability. This method facilitates direct comparisons between different oils and antioxidants by eliminating variations due to inconsistent measurement conditions (Suhag, Razem, et al. 2024).

Despite these advantages, DPC has certain limitations. While it provides detailed kinetic data, complementary techniques are often required to identify oxidation products formed during the reaction. Moreover, the small sample volume used in DPC experiments may not be sufficient for post‐irradiation analyses, potentially limiting additional characterization. Another constraint is that only one sample can be analyzed at a time, making the process time‐consuming and limiting throughput for large‐scale studies (Suhag, Razem, et al. 2024).

## 4 Practical Relevance of Kinetic‐Based Methods

Although kinetic‐based antioxidant testing methods provide mechanistic and time‐resolved insights, their industrial adoption remains limited, and they remain predominantly confined to research and development. Nevertheless, increasing demand for real‐time antioxidant evaluation, particularly in food, nutraceutical, and cosmetic industries, highlights their promise for future applications. The stopped‐flow DPPH kinetic method, for instance, enables rapid assessment of antioxidant reactivity and reaction mechanisms, making it invaluable for studying fast‐acting compounds like polyphenols. Despite its current restriction due to complex instrumentation, simplified or automated versions could transform it into a high‐resolution screening tool for supplement and functional beverage development. In contrast, the ORAC kinetic assay leverages widely accessible microplate readers, aligning with routine laboratory workflows. Although kinetic modeling of ORAC data still remains at academic level, its application to functional beverages or botanical extracts could allow differentiation of antioxidants by reactivity and repair potential, rather than total capacity alone.

Isothermal calorimetry, already utilized in pharmaceutical and material sciences industries, offers high throughput with the ability to process several samples simultaneously and compatibility with real food matrices. Scalability for antioxidant screening in product development, ingredient selection, and quality control underscores its versatility across sectors like dairy and bakery. Oxygen uptake measurement remains a precise approach for studying lipid oxidation in complex systems, despite limitations in throughput. Its accuracy justifies its use in high‐value applications, such as validating antioxidant efficacy in premium oils or cosmetics. Similarly, DPC simulates light‐induced oxidative stress and holds experimental potential for quality assessments of light‐sensitive products like edible oils or plant‐based packaging materials, particularly with standardized protocols for real‐world storage conditions.

Emerging techniques, like fluorescent probe monitoring (e.g., STY‐BODIPY), are gaining attraction for the high‐throughput oxidation evaluation in food oils. Compatible with standard fluorescence readers, this method enables rapid screening of antioxidant reactivity in food matrices, with prospective roles in ingredient optimization, formulation testing, and batch comparisons for oils, seasonings, or fortified products. Collectively, these methods, though largely applied at academic level, offer distinct advantages aligning with industrial needs. High‐throughput approaches such as isothermal calorimetry and fluorescent probe monitoring are poised for integration into routine workflows, whereas specialized methods like oxygen uptake and photocalorimetry provide precision for validating complex systems. As industries increasingly prioritize mechanism‐based evaluation, these techniques will likely transition from academic application to mainstream practice, tailoring specific requirements needing for accuracy and efficiency. Table 3 summarizes the comparative analysis of kinetic‐based methods.

**TABLE 3:** Comparative analysis of different kinetic‐based methods.

Method	Real food matrix compatibility	Throughput	Time required per experiment	Instrument prevalence in standard labs	Scope of practical application
Stopped‐flow DPPH kinetic method	Low; suitable for purified antioxidants/extracts in solvent systems	Low; single sample can be analyzed at once	Seconds to minutes	Moderately available and is commercially available	Rapid screening and reactivity profiling of purified antioxidants/extracts with potential in nutraceutical/food industry for quality control and standardization of antioxidant ingredients
ORAC kinetics	Moderate; suitable for aqueous food products such as juices, tea, functional beverages	High with the use of 96‐well plate	Minutes to hours	Widely available (fluorescence plate reader)	Useful for functional beverage manufacturers to validate label claims and compare radical scavenging ability
Oxidizable substrate monitoring	Moderate; suitable for oils and clear emulsions; may cause interference in complex food products	High with the use of 96‐well plate	Minutes to hours	Widely available (fluorescence plate reader) but needs specialized fluorescent probe	Useful for vegetable oil producers to estimate the ability of synthetic and natural antioxidants in improving oxidative stability
Oxygen uptake method	High; suitable for a range of food products	Low; single sample can be analyzed at once	Minutes to hours	Specialized setup not common and not commercially available	Useful for food and cosmetic industries to determine the oxidative stability and antioxidant testing in a variety of food products
Isothermal calorimetry	High; suitable for a range of food products	High; upto 48 samples can be analyzed simultaneously	Hours to days	Low availability, but is commercially available	Useful for food manufacturers for shelf‐life prediction, batch‐to‐batch consistency, and quality control
Differential photocalorimetry	High; suitable for a range of food products	Low; single sample can be analyzed at once	Hours to days	Widely available, but needs a light source that is commercially available	Potential for simulating photo‐oxidative stability in food products and light‐exposed skincare products. Moreover, it can be useful in the development of UV‐protective packaging

Abbreviations: DPPH, 1,1‐diphenyl‐2‐picrylhydrazyl; ORAC, oxygen radical absorbance capacity.

## 5 Conclusion and Future Perspectives

The assessment of antioxidants has traditionally relied on quantitative measurements, such as total phenolic content and antioxidant capacity, which provide a broad overview of antioxidant potential. However, these approaches often failed to capture the kinetic aspects of antioxidant activity, which are crucial for understanding their effectiveness in complex food systems. The shift toward kinetic‐based antioxidant testing represents a significant advancement, offering insights into reaction rates and stoichiometry under realistic oxidative conditions. Methods such as isothermal calorimetry, oxygen uptake, and DPC have emerged as powerful tools for evaluating antioxidant reactivity, allowing a more precise assessment of their protective role in food systems.

Despite these advancements, these methods are still in their initial phases and require continuous refinement before they can emerge as standardized kinetic‐based approaches for antioxidant testing. Additionally, although these approaches provide deeper mechanistic insights, they often require specialized instrumentation and longer experimental durations compared to conventional assays. Addressing these challenges will necessitate harmonized protocols, greater accessibility of analytical tools, and further integration of computational modeling to enhance predictive capabilities.

The evolution of antioxidant assessment demands a cohesive, multi‐branched strategy that integrates rigorous method validation, mechanistic insights into reactive oxygen species (ROS) biology, and cutting‐edge technological innovation. Central to this approach is the standardization of analytical protocols through collaborative inter‐laboratory validation, which reduces variability and ensures reproducibility across diverse matrices (Amft et al. 2023). For example, Breusing et al. (2010) underscored the importance of such validation in lipid peroxidation assays, resolving inconsistencies in malondialdehyde (MDA) quantification through standardized calibration curves and matrix‐matched controls. Additionally, to achieve regulatory compliance, kinetic antioxidant methods must first demonstrate reproducibility and robustness by adhering to performance criteria such as precision, accuracy, and reproducibility, as outlined in AOAC's SMPR 2011.011 (Oxford Academic 2012). Furthermore, health claim substantiation requires that kinetic constants reliably correlate with *in* *vivo* biomarkers of oxidative damage, as per EFSA's guidance on antioxidants and oxidative damage (Turck et al. 2018).

Emerging insights into ROS dynamics highlight their dual roles as physiological mediators and pathological drivers, necessitating advanced antioxidant screening. Excessive ROS production disrupts redox homeostasis, fueling chronic inflammation and carcinogenesis via NF‐κB/MAPK pathways (Guo, Jin, et al. 2024). For example, oxidized olive oil generates pro‐inflammatory lipid peroxidation products (e.g., 9,10‐epoxy‐stearic acid) that impair mitochondrial function and amplify intestinal/hepatic inflammation (Bao et al. 2025), whereas fish oil *n*‐3 fatty acids induce ROS‐dependent ferroptosis in protumor macrophages, uncoupling obesity from mammary tumor growth (Liu et al. 2020). Integrating oxidomics systematic profiling of oxidative stress biomarkers reveals ROS interactions with lipids (e.g., ceramides, 4‐HNE), proteins, and DNA, linking redox imbalance to inflammatory diseases (Jia et al. 2023). AI‐driven lipidomics identifies dysregulated pathways (e.g., arachidonic acid signaling) and predicts antioxidant efficacy, as shown in TNF‐α‐induced inflammatory models (Aiello et al. 2024). This approach, combined with kinetic data, can enable specific antioxidant strategies to modulate ROS pathways without disrupting essential redox signaling.

Alongside, AI‐driven platforms are revolutionizing antioxidant discovery by predicting synergistic interactions (Ayres et al. 2023). Machine learning (ML) models, including graph convolutional networks and random forests, can help identify antioxidants with enhanced stability in lipid systems (Pantic et al. 2022). Finally, regulatory agencies must adopt adaptive frameworks that accommodate rapid technological advances, such as robotics‐driven high‐throughput screening and lab‐on‐a‐chip systems for real‐time antioxidant monitoring.

By unifying method validation, oxidomics insights, and AI‐augmented discovery, the scientific community can transition from static antioxidant quantification to dynamic, application‐specific strategies. Sustained investment in automation, regulatory alignment, and cross‐sector collaboration will solidify kinetic approaches as gold standards, advancing both food preservation and therapeutic interventions in oxidative stress‐related diseases.

## Author Contributions

**Rajat Suhag**: conceptualization, investigation, methodology, writing – original draft, visualization. **Lucrezia Angeli**: conceptualization, methodology. **Matteo Scampicchio**: conceptualization, formal analysis. **Giovanna Ferrentino**: conceptualization, supervision, project administration, writing – review and editing.

## Conflicts of Interest

The authors declare no conflicts of interest.
